# How the Chalcogen Atom Size Dictates the Hydrogen‐Bond Donor Capability of Carboxamides, Thioamides, and Selenoamides

**DOI:** 10.1002/chem.202200755

**Published:** 2022-04-26

**Authors:** Celine Nieuwland, Célia Fonseca Guerra

**Affiliations:** ^1^ Department of Theoretical Chemistry Amsterdam Institute of Molecular and Life Sciences (AIMMS) Amsterdam Center for Multiscale Modeling (ACMM) Vrije Universiteit Amsterdam De Boelelaan 1083 1081 HV Amsterdam The Netherlands; ^2^ Leiden Institute of Chemistry Gorlaeus Laboratories Leiden University Einsteinweg 55 2333 CC Leiden The Netherlands

**Keywords:** amides, chalcogens, density functional calculations, hydrogen bonding, organocatalysis

## Abstract

The amino groups of thio‐ and selenoamides can act as stronger hydrogen‐bond donors than of carboxamides, despite the lower electronegativity of S and Se. This phenomenon has been experimentally explored, particularly in organocatalysis, but a sound electronic explanation is lacking. Our quantum chemical investigations show that the NH_2_ groups in thio‐ and selenoamides are more positively charged than in carboxamides. This originates from the larger electronic density flow from the nitrogen lone pair of the NH_2_ group towards the lower‐lying π*_C=S_ and π*_C=Se_ orbitals than to the high‐lying π*_C=O_ orbital. The relative energies of the π* orbitals result from the overlap between the chalcogen *n*p and carbon 2p atomic orbitals, which is set by the carbon‐chalcogen equilibrium distance, a consequence of the Pauli repulsion between the two bonded atoms. Thus, neither the electronegativity nor the often‐suggested polarizability but the *steric size* of the chalcogen atom determines the amide's hydrogen‐bond donor capability.

## Graphical abstract

**The bigger the better**! Thioamides and selenoamides are better hydrogen‐bond donors than carboxamides because their amino groups are more positively charged. Quantum chemical analyses reveal that this counterintuitive phenomenon, which is not explainable by the electronegativity, results from the effective steric size of the chalcogen atoms.

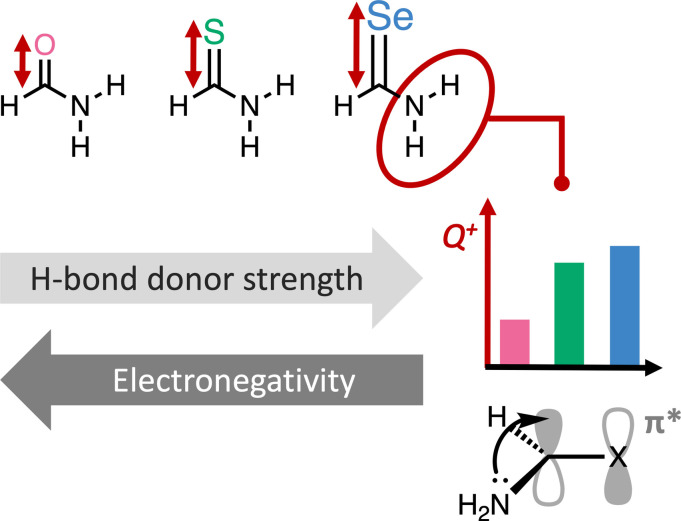

## Introduction

The use of non‐covalent organocatalysts has emerged as a powerful catalytic method in asymmetric organic synthesis.^[1]^ By creating enzyme‐like catalytic sites, chemical transformations can occur with high proficiencies and selectivities. Within enzymes, the catalytic activity is often governed by forming hydrogen‐bond interactions with the substrate. Therefore, novel organocatalysts often employ the assembly of catalytic species, connected through multiple hydrogen bonds. Especially, bifunctional hydrogen‐bond donor amide organocatalysts, such as ureas and squaramides, have attracted considerable attention in this field.^[2]^ These bidentate organocatalysts preorganize and activate hydrogen‐bond accepting substrates, leading to enhanced selectivities and reactivities among a large scope of organic reactions. Thioamides are intrinsically more acidic than the oxygen analogs, thereby strengthening the hydrogen‐bond interaction with the substrate, and may therefore be more attractive as organocatalysts.^[3]^ However, a sound electronic explanation of this enhanced hydrogen‐bond donor strength is lacking. A recent study by Vermeeren et al. explaining the catalysis of Diels‐Alder reactions by urea and thiourea catalysts showed that thioureas indeed form stronger hydrogen‐bonded complexes with substrates leading to a higher catalytic effect.^[4]^ The enhanced hydrogen‐bond donor strength was found to be the consequence of more stabilizing electrostatic and orbital interactions in the thiourea hydrogen‐bonded complexes, compared to the urea analogs. These results are in contradiction with the widely accepted rationale, from which one would expect stronger hydrogen bonds for carboxamides considering that the electronegativity of the chalcogens decreases significantly from O to S and as a consequence, more stabilizing electrostatic interactions for carboxamides.

The exploitation of the enhanced hydrogen‐bond strengths for amides containing the heavier chalcogens extends to the field of supramolecular polymer chemistry as recently it became evident that the replacement of oxygen in the amide bond for the heavier chalcogens, sulfur and selenium, can produce supramolecular polymers with similar or even stronger hydrogen bonds.^[5]^ As the arguments based on the electronegativity differences of O, S, and Se cannot rationalize the enhanced hydrogen‐bond strength for amides comprising heavier chalcogens, most studies use explanations in terms of increasing polarizability and charge capacity.[^5a^, ^6^] Using an oversimplified resonance model, the highly polarizable heavy chalcogen atoms would be able to carry more negative charge. However, the causal relationship between the chalcogen's polarizability and the hydrogen‐bond strength is not proven and no clear insights are provided into how to tune the hydrogen‐bond donor capabilities of amides.

**Figure 1:**
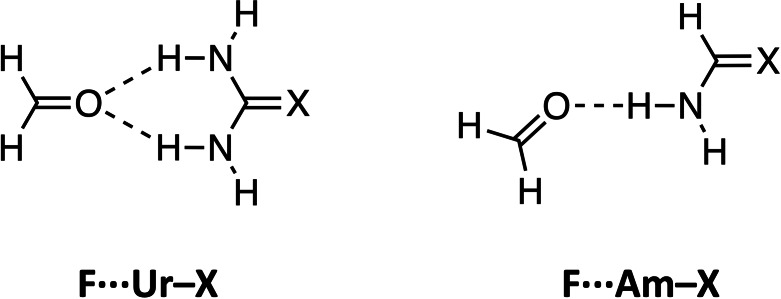
The hydrogen‐bonded complexes studied in this work with X=O, S, and Se: formaldehyde‐chalcourea (**F⋅⋅⋅Ur**−**X**) and formaldehyde‐chalcoamide (**F⋅⋅⋅Am**−**X**).

In this work, we trace the origin of the enhanced hydrogen‐bond donor strengths of amides containing the heavier chalcogens S and Se by studying the hydrogen‐bond interaction of bidentate chalcourea (**Ur**−**X**, with X=O, S, and Se), and monodentate chalcoamide (**Am**−**X**) hydrogen‐bond donors with a carbonyl substrate, formaldehyde (**F**) (see Figure 1).

Formaldehyde was chosen as the substrate as (thio)urea catalysts are often employed in carbonyl activation.^[2]^ For our analyses, we performed relativistic dispersion‐corrected Density Functional Theory (DFT−D) computations at ZORA‐BLYP‐D3(BJ)/TZ2P to explain the hydrogen bonding abilities of the different amides in the framework of Kohn‐Sham molecular orbital theory. First, we examine the hydrogen‐bonding interactions by the state‐of‐the‐art computational method: the activation strain model (ASM)^[7]^ of reactivity and bonding, and reproduce trends in line with experiment. Further decomposition of the hydrogen‐bond energy into fundamental terms,^[8]^ shows that the enhanced amide hydrogen‐bond donor strength for the heavier chalcogens, caused by more favorable orbital and electrostatic interactions, is the consequence of positive charge accumulation on the amino (NH_2_) groups. In the next step, by performing an extensive bonding analysis on the amide molecules, we demonstrate that the different degree of charge accumulation originates from the nature of the C=X antibonding π* orbital. In the final part, we analyze the construction of this π*_C=X_ orbital from the p atomic orbitals of carbon and the chalcogen atoms, leading to new insights into the nature of hydrogen bonding involving amides for the development of novel hydrogen‐bonded materials and organocatalysts.

## Results and Discussion

### Hydrogen‐bond energies and geometries

To gain insight into the nature of hydrogen bonding of amides containing various chalcogens, the **F⋅⋅⋅Ur**−**X** and **F⋅⋅⋅Am**−**X** hydrogen‐bonded complexes (Figure 1, for X=O, S, and Se) were examined by using dispersion‐corrected relativistic density functional theory (DFT−D) computations at the ZORA‐BLYP‐D3(BJ)/TZ2P level of theory in the gas phase using the Amsterdam Density Functional (ADF) program (see Supporting Information Method S1 for full computational details).[^9^, ^10^, ^11^, ^12^] This level of theory has been proven to be accurate for the description of hydrogen‐bond interactions, both in previous reports,^[13]^ as well as in the computational performance tests carried out in the present work (see Supporting Information Method S1 for details, and Table S1‐S3 for the results).

**F⋅⋅⋅Ur**−**X** are complexes containing two hydrogen bonds, the so‐called bifurcated hydrogen bonds, whereas the **F⋅⋅⋅Am**−**X** complexes comprise a single hydrogen bond. For the **F⋅⋅⋅Am**−**X** complexes we found a *C*_1_ (non‐planar) and a *C*_s_ symmetric (planar) minimum (i. e., no imaginary frequencies) that are very close in energy (see the Supporting Information for the Cartesian coordinates and total bond energies of the optimized structures). For each of the **F⋅⋅⋅Ur**−**X** complexes we identified one *C*_1_ minimum, but we obtained through a constrained optimization analogous *C*_2v_ symmetric structures that are close in energy and display only imaginary frequencies associated with the pyramidalization of the amino groups. In our analyses, we focus primarily on the *C*_2v_ **F⋅⋅⋅Ur**−**X** and *C*_s_ **F⋅⋅⋅Am**−**X** complexes, to which we refer as “planar complexes”. These planar structures furnish identical hydrogen‐bond energy trends as the non‐planar *C*_1_ complexes (see Tables S2 and S3). The use of planar geometries allows for the separation of interactions within the σ and π electronic system which will become useful later on in this work.[^13b^, ^13f^, ^14^, ^15^]

**Figure 2:**
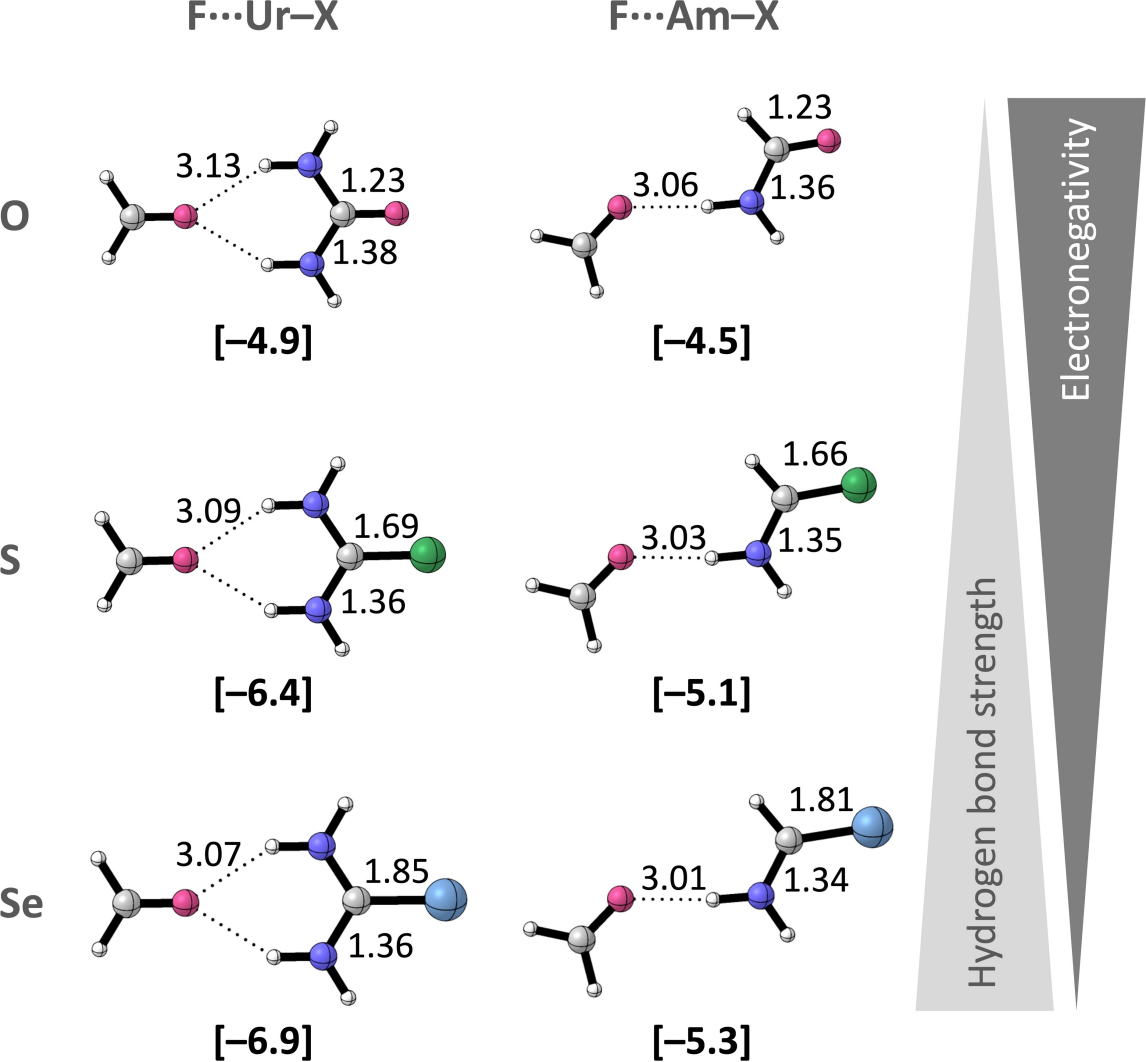
Equilibrium hydrogen‐bond (O⋅⋅⋅(H)N), C=X, and C−N distances (in Å) for the **F⋅⋅⋅Ur**−**X** and **F⋅⋅⋅Am**−**X** planar complexes with X=O, S, and Se. Hydrogen‐bond energies Δ*E*_bond_ (in kcal mol^−1^) are shown below the structures between brackets. Color code of the ball‐and‐stick structures: hydrogen=white; carbon=gray; nitrogen=dark blue; oxygen=pink; sulfur=green; selenium=light blue.

The planar optimized hydrogen‐bonded complexes are presented in Figure 2, alongside the calculated hydrogen‐bond energies (Δ*E*_bond_) and relevant bond distances (see Figure S3 for the non‐planar complexes). Figure 2 shows that Δ*E*_bond_ becomes more stabilizing along the trend X=O<S<Se, which is also reflected by the shortening of the hydrogen bond (O⋅⋅⋅(H)N) and is in line with previous experimental and computational reports.[^2^, ^4^, ^5^] To understand the different components that determine the trend in hydrogen‐bond strength, Δ*E*_bond_ was partitioned into the strain (Δ*E*_strain_) and the interaction energy (Δ*E*_int_) according to the activation strain model (ASM) of reactivity and bonding (Eq. (1), see Supporting Information Method S2 for details).^[7]^

(1) $$\Delta E{}_{\mathrm{bond}}=\Delta E{}_{\mathrm{strain}}+\Delta E{}_{\mathrm{int}}$$

Δ*E*_strain_ is the energy required to deform each monomer from its equilibrium geometry to the geometry it acquires when it interacts in the hydrogen‐bonded complex. For the *C*_1_ complexes, Δ*E*_strain_ is small and roughly equal for all chalcogens (Figure S4 and Table S2). In the planar complexes, Δ*E*_strain_ amounts 0.1 kcal mol^−1^ for all **F⋅⋅⋅Am**−**X** complexes, and 1.1, 0.5, and 0.3 kcal mol^−1^ for the **F⋅⋅⋅Ur**−**X** complexes with X=O, S, and Se, respectively (Table S3). In the latter complexes, the decreasing strain is associated with the decreasing pyramidalization of the NH_2_ groups in the **Ur**−**X** equilibrium geometries going from X=O to S to Se, with **Ur**−**Se** being close to planar (see Figure S5). The **Am**−**X** and **F** equilibrium geometries are all planar.

**Figure 3:**
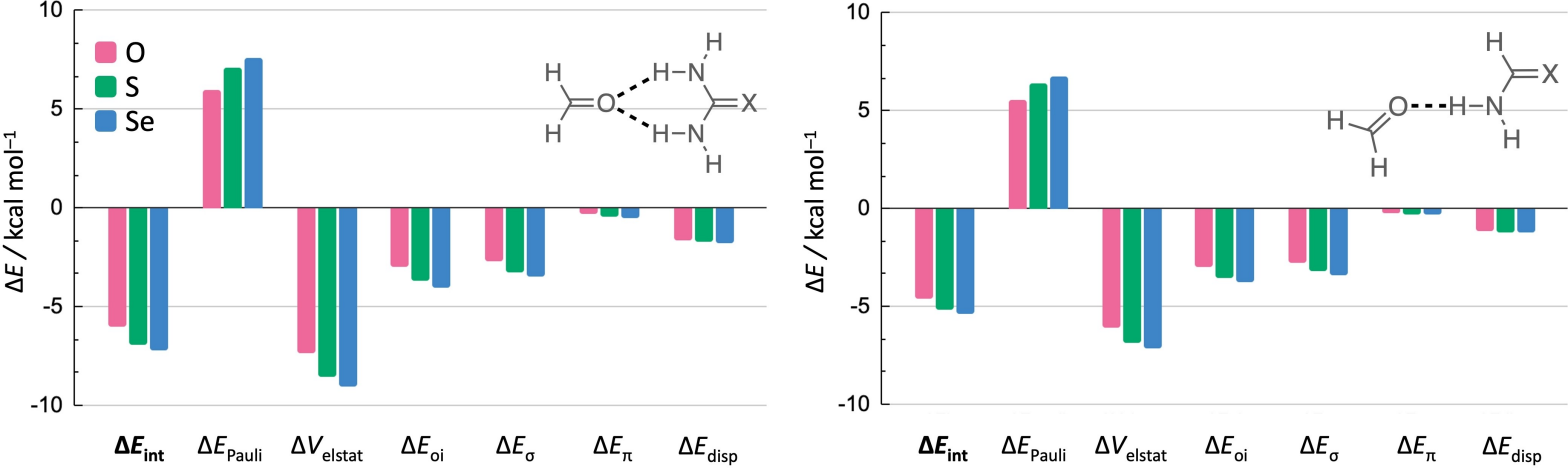
Decomposition of the interaction energy Δ*E*_int_ (in kcal mol^−1^) of the hydrogen‐bond interaction in the **F⋅⋅⋅Ur**−**X** (left) and **F⋅⋅⋅Am**−**X** (right) planar complexes with X=O, S, and Se.

In Equation (1), Δ*E*_int_ accounts for the net stabilizing interaction between the two prepared (i. e., deformed) monomers. For both the non‐planar and planar complexes (Tables S2 and S3) it was found that the stabilizing trend of the hydrogen‐bond energy when moving down Group 16 is the result of the Δ*E*_int_ component which follows the same stabilizing trend (Figure 3).

As the interaction energy determines the trend of the hydrogen‐bond energy, we decomposed Δ*E*_int_ into physically meaningful terms, as formulated in Equation (2) (see Supporting Information Method S2 for details).^[8]^

(2) $$\Delta E{}_{\mathrm{int}}=\Delta E{}_{\mathrm{Pauli}}+\Delta V{}_{\mathrm{elstat}}+\Delta E{}_{\mathrm{oi}}+\Delta E{}_{\mathrm{disp}}$$

This quantitative energy decomposition analysis (EDA) based on Kohn‐Sham molecular orbital theory, divides the total interaction energy (Δ*E*_int_) into Pauli repulsion (Δ*E*_Pauli_), electrostatic interaction (Δ*V*_elstat_), orbital interaction (Δ*E*_oi_), and dispersion (Δ*E*_disp_) energy components. Δ*E*_Pauli_ comprises the destabilizing interactions arising from overlapping occupied orbitals and accounts for any steric repulsion. Δ*V*_elstat_ corresponds to classical electrostatic interactions between the unperturbed charge distributions of the prepared (i. e., deformed) interacting molecular fragments and is usually attractive. The term Δ*E*_oi_ includes charge transfer (i. e., donor‐acceptor interactions between occupied orbitals on one of the interacting fragments and unoccupied orbitals on the other, including HOMO‐LUMO interactions), polarization (empty‐occupied orbital mixing on one fragment due to the presence of the other fragment), and electron‐pair interactions (e. g., SOMO‐SOMO interactions). The Δ*E*_disp_ term includes a dispersion energy correction. Lastly, since we analyze planar complexes, the orbital interaction term (Δ*E*_oi_) can be decomposed into the contributions of the σ (Δ*E*_σ_) and π (Δ*E*_π_) orbitals (Eq. 3).

(3) $$\Delta E{}_{\mathrm{oi}}=\Delta E{}_{\sigma }+\Delta E{}_{\pi }$$

From the EDA results in Figure 3 follows that the stabilizing trend in Δ*E*_int_ from X=O to S to Se, and thus the enhanced hydrogen‐bond donor strength, results mainly from the electrostatic interaction (Δ*V*_elstat_), and orbital interaction (Δ*E*_oi_) to a lesser extent as both interaction terms become more stabilizing for the heavier chalcogens. This is in line with the results found by Vermeeren and co‐workers.^[4]^ The Δ*E*_oi_ term arises primarily from orbital interactions within the σ system (Δ*E*_σ_) and from a small contribution of the π system (Δ*E*_π_), which is in accordance with previous research into the nature of hydrogen bonds.[^15^, ^16^] The dispersion and Pauli repulsion do not contribute to the strengthening of the hydrogen bonds as Δ*E*_disp_ becomes only slightly more stabilizing and Δ*E*_Pauli_ becomes more *destabilizing* for the heavier chalcogens. Both effects are associated with the shorter hydrogen bond in the thio‐ and selenoamide complexes (Figure 2).

### Origin of the enhanced hydrogen‐bond donor strength

The enhanced orbital and electrostatic interactions in the hydrogen bonding are responsible for the better hydrogen‐bond donor capability of thioamides and selenoamides compared to carboxamides. By performing an extensive analysis on both interaction terms in this section, we aim at revealing the origin of this counterintuitive observation according to the electronegativity differences of the chalcogens.

**Figure 4:**
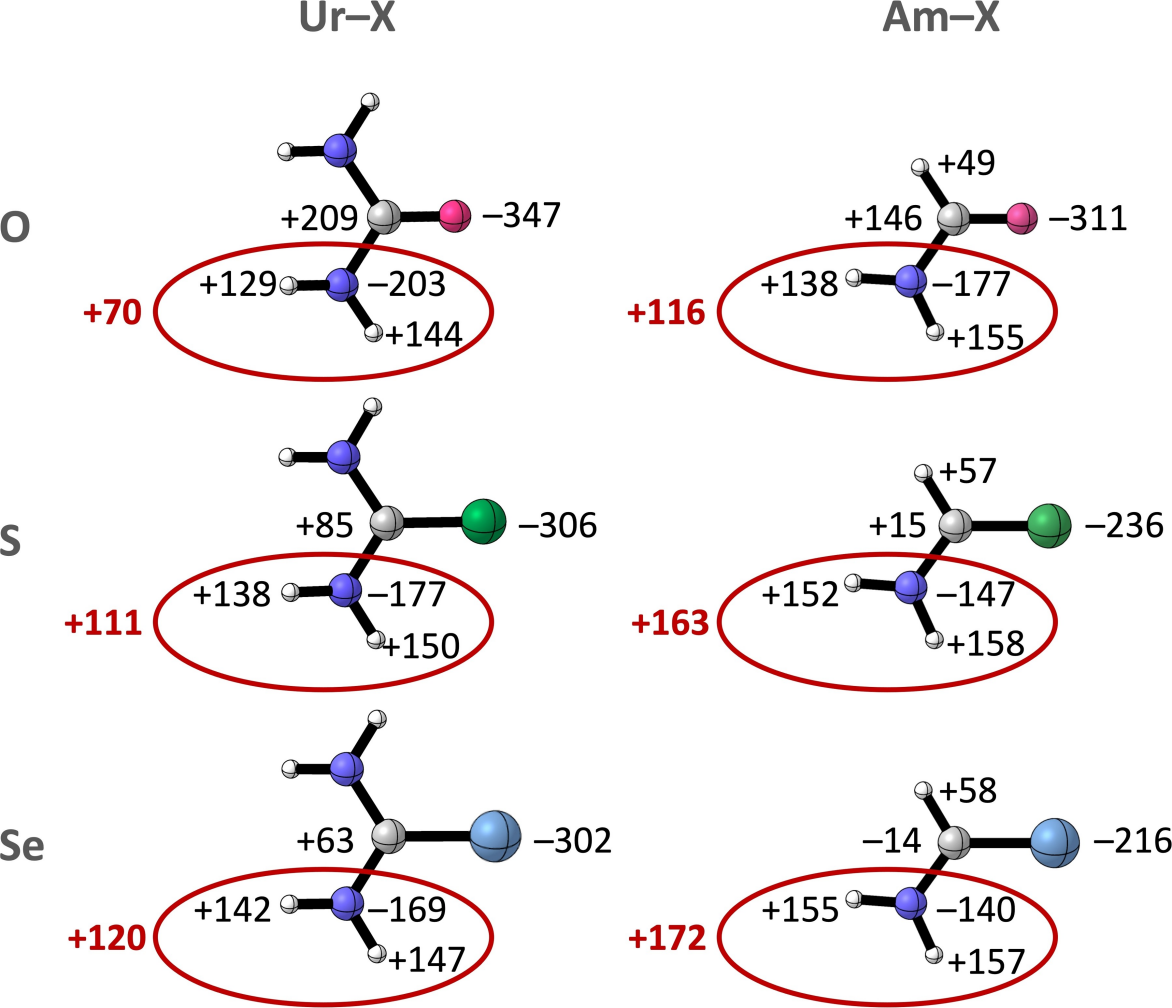
VDD atomic charges *Q* (in milli‐electrons) in the equilibrium geometries of **Ur**−**X** and **Am**−**X** with X=O, S, and Se. The total *Q* on the NH_2_ group is shown in red. Color code of the ball‐and‐stick structures: hydrogen=white; carbon=gray; nitrogen=dark blue; oxygen=pink; sulfur=green; selenium=light blue.

First, we explain the role of the electrostatics (Δ*V*_elstat_) in the enhanced hydrogen‐bond donor strength of thio‐ and selenoamides by assessing the Voronoi deformation density (VDD)^[14]^ atomic charges (*Q*, see Supporting Information Method S3 for computational details) of the **Ur**−**X** and **Am**−**X** equilibrium geometries in Figure 4 and demonstrate that the amino groups are responsible for the observed trend in Δ*V*_elstat_.

We analyze here the equilibrium geometries of the free amide monomers as we want to arrive at a unified framework for understanding the hydrogen‐bond donor abilities of amides containing chalcogens, regardless of the type of hydrogen‐bond acceptor.

In **Ur**−**X** and **Am**−**X** from X=O to S to Se, we see that the nitrogen atom becomes less negatively charged and the hydrogen atoms more positively charged, overall leading to more positive NH_2_ groups in amides containing S or Se (shown in red in Figure 4). More positive NH_2_ groups naturally lead to a more stabilizing Δ*V*_elstat_ within the hydrogen‐bonded complex with **F**, and thus stronger hydrogen bonds for the thio‐ and selenoamides (see above). These results are in contradiction with the commonly accepted view, from which one would expect the amino groups of carboxamides to carry the most positive charge by both inductive and resonance effects, based on the highest electronegativity of oxygen. In line with the decrease in electronegativity from O to S to Se is the VDD charge on the chalcogen atoms, where the most electronegative element O also carries the most negative charge, while the less electronegative elements S and Se are less negatively charged, and the carbon atom becomes less positive going from X=O to S to Se.

**Figure 5:**
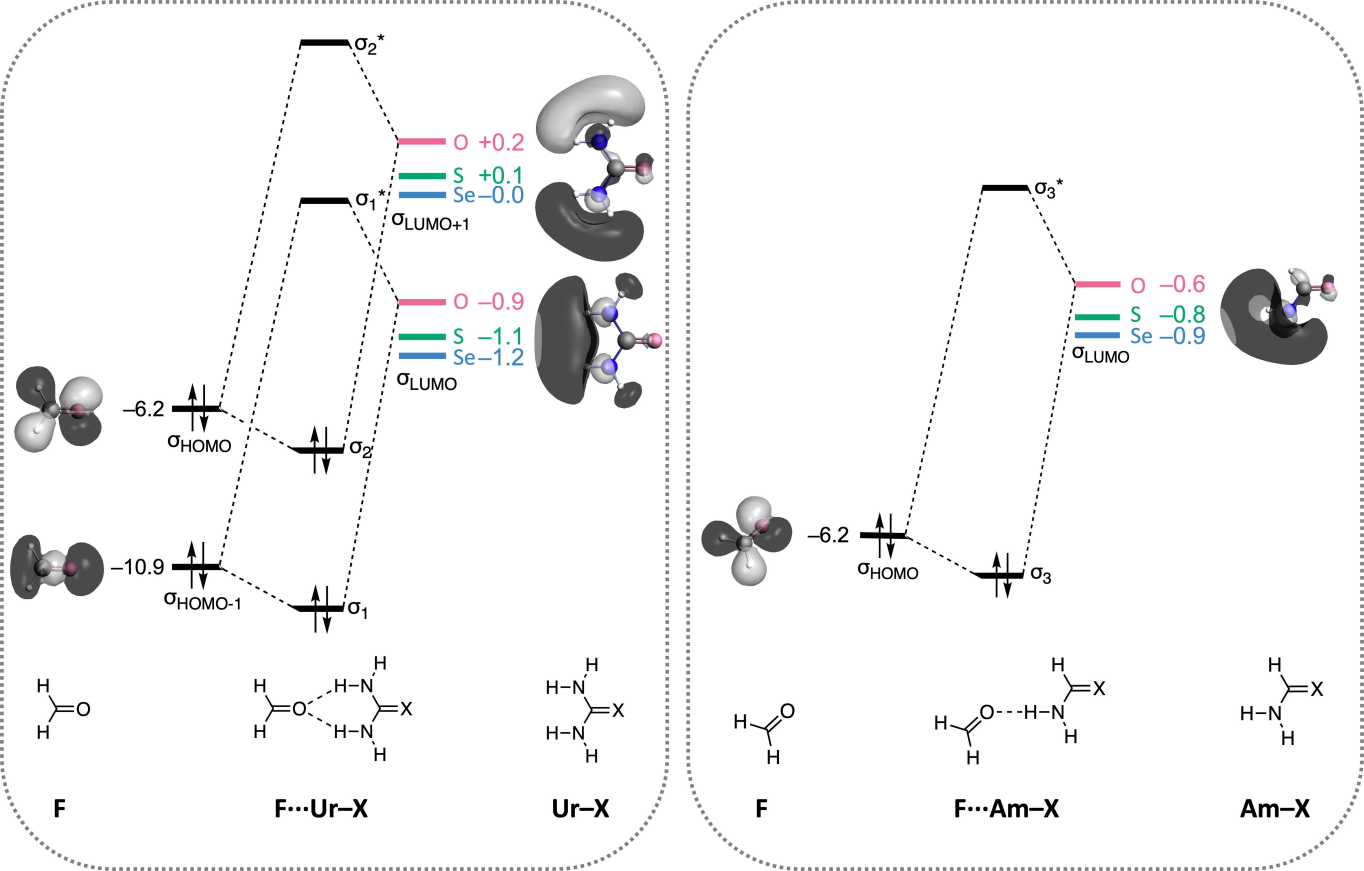
Schematic molecular orbital (MO) interaction diagram for the hydrogen‐bond interaction in the **F⋅⋅⋅Ur**−**X** (left) and **F⋅⋅⋅Am**−**X** (right) planar complexes with X=O, S, and Se. The orbital energies (in eV), and MO isosurfaces (at 0.03 au) for the carboxamide complexes are shown.

In addition to Δ*V*_elstat_, the orbital interaction (Δ*E*_oi_) becomes more stabilizing going from X=O to Se, contributing to the enhanced hydrogen‐bond donor strengths for thio‐ and selenoamides (Figure 3). To clarify this effect, an orbital interaction diagram was constructed for the molecular orbitals (MOs) involved in the hydrogen‐bonding interaction with formaldehyde. The schematic MO diagrams for the **F⋅⋅⋅Ur**−**X** and **F⋅⋅⋅Am**−**X** complexes are presented in Figure 5. For the corresponding MO energies, gross Mulliken populations, and orbital overlaps, we refer the reader to Tables S4 and S5.

Figure 5 shows that the lowest unoccupied molecular orbital (σ_LUMO_) levels for the amides containing the heavier chalcogens are energetically lower than the corresponding σ_LUMO_ of carboxamides. The σ_LUMO_ of thio‐ and selenoamides is stabilized compared to the σ_LUMO_ of carboxamides due to the larger positive charge on the amino groups (see above). This translates into a more stabilizing Δ*E*_oi_ for thio‐ and selenoamides as the HOMO‐LUMO gap with the highest occupied molecular orbital (σ_HOMO_) of formaldehyde decreases. The orbital overlap remains nearly constant for all systems (see Table S4).

The bifurcated hydrogen bonds in the **F⋅⋅⋅Ur**−**X** complex arise from the donor‐acceptor interactions of the σ_HOMO‐1_ and σ_HOMO_ of **F**, with respectively the σ_LUMO_ and σ_LUMO+1_ of **Ur**−**X**. Both the σ_LUMO+1_ and σ_LUMO_ levels are lowered in energy from X=O to Se, resulting in a stronger mixing with the lower‐lying HOMOs of **F** in the case of thio‐ and selenourea.

In the **F⋅⋅⋅Am**−**X** complex, only one hydrogen bond is formed, which arises from the donor‐acceptor interaction between the σ_HOMO_ of **F** with the σ_LUMO_ of **Am**−**X**. Again, lowering of the amide's σ_LUMO_ is observed going from carboxamide (**Am**−**O**) to thioamide (**Am**−**S**) to selenoamide (**Am**−**Se**) due to the increase of positive charge on NH_2_ and explains the more stabilizing orbital interactions along this trend.

### Explaining the positive charge accumulation on the NH_2_ groups

**Figure 6:**
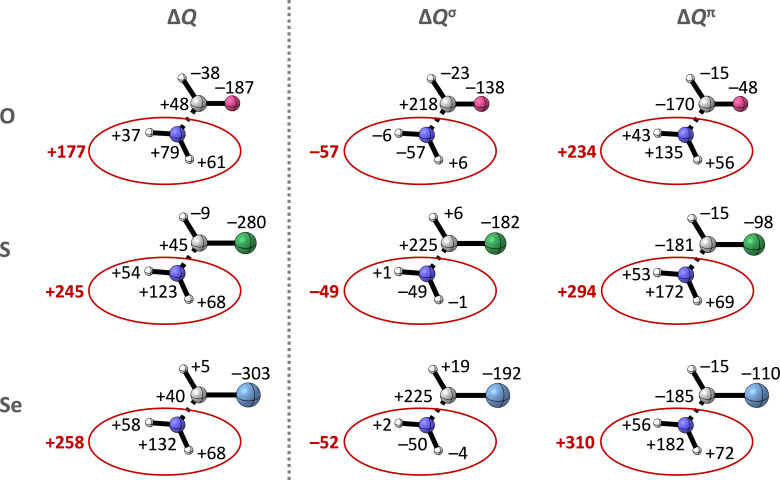
Change in VDD atomic charge Δ*Q* (in milli‐electrons) and contributions from the σ (Δ*Q* ^σ^) and the π electrons (Δ*Q* ^π^), upon forming the σ_C−N_ bond between the ⋅NH_2_ and ⋅(H)C=X fragment in the **Am**−**X** equilibrium geometries with X=O, S, and Se. The total Δ*Q* of the NH_2_ group is shown in red. Color code of the ball‐and‐stick structures: hydrogen=white; carbon=gray; nitrogen=dark blue; oxygen=pink; sulfur=green; selenium=light blue.

To trace the origin of the positive charge accumulation on the amino groups of the amides comprising the heavier chalcogens, the change in VDD atomic charge Δ*Q* upon forming the amide bond (σ_C−N_) in the **Am**−**X** equilibrium geometries was computed and is presented in Figure 6 (see Supporting Information Method S3 for computational details).^[14]^ The formation of this covalent bond leads to a net flow of electrons (Δ*Q*) from the NH_2_ group to the (H)C=X fragment, leading to the accumulation of positive charge on the NH_2_ group (shown in red in Figure 6). This electronic density flow increases from X=O to S to Se, confirming that the NH_2_ groups become increasingly positive in amides containing heavier chalcogens.

The total charge redistribution Δ*Q* can be split into contributions of the σ (Δ*Q* ^σ^) and π electrons (Δ*Q* ^π^), accounting for charge transfer within the σ and π electronic system (see Figure 6). Δ*Q* ^σ^ shows for the three amides donation of σ electrons *to* the NH_2_ groups (−57, −49, and −52 milli‐electrons for X=O, S, and Se, respectively), and therefore the electron depletion on the NH_2_ groups does not occur within the σ system. Δ*Q* ^π^ on the other hand shows a large electron density flow *from* the NH_2_ group to the (H)C=X fragment which increases going from **Am**−**O** to **Am**−**Se** (+234, +294, and +310 milli‐electrons for **Am**−**O**, **Am**−**S**, and **Am**−**Se**, respectively). So, the accumulation of positive charge on the amide NH_2_ groups originates from charge shifts within the π system upon forming the C−N bond.

**Figure 7:**
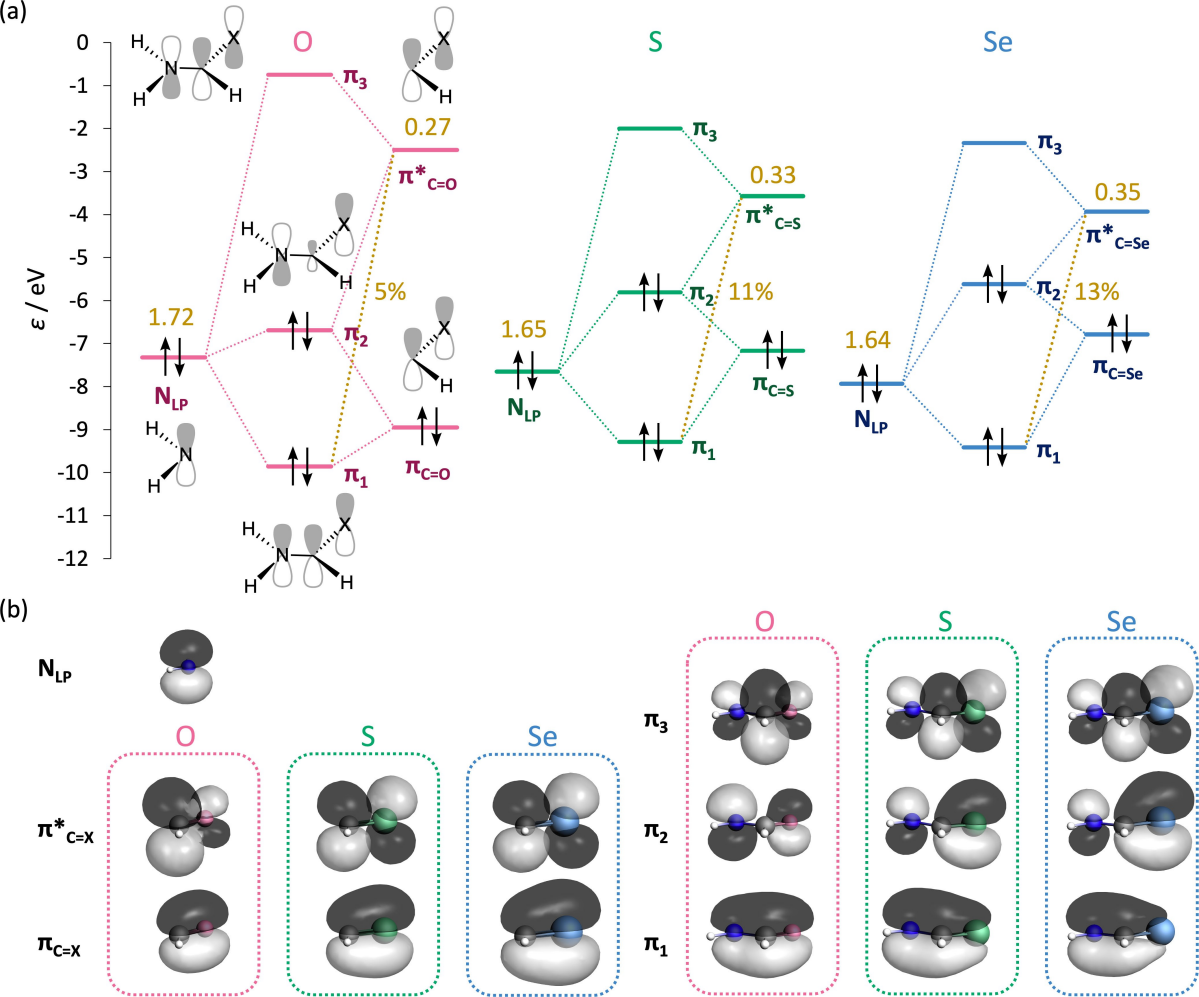
a) Orbital interaction diagram for the π interaction upon forming the C−N bond in the **Am**−**X** equilibrium geometries with X=O, S, and Se. Gross Mulliken populations (in electrons) and contributions of π*_C=X_ to the bonding π_1_ MO (in %) are highlighted in yellow. b) Visualization of the orbitals involved in the π interaction (isosurfaces at 0.03 au).

To explain this in more detail, a qualitative MO analysis of the π interaction between the ⋅NH_2_ and ⋅(H)C=X fragments in **Am**−**X** was performed and is presented in Figure 7 (see Table S6 for the numerical values and Table S7 for the energy decomposition terms). This interaction comprises a repulsive interaction (i. e., Pauli repulsion) between the nitrogen lone pair‐type p orbital (N_LP_) and the filled π bonding orbital of the C=X bond (π_C=X_), and a stabilizing donor‐acceptor interaction between N_LP_ and the empty antibonding π* orbital of the C=X bond (π*_C=X_).

From Figure 7a follows that the π*_C=X_ orbital is responsible for different degrees of charge transfer within the π system for the various **Am**−**X** molecules. The π* level of the ⋅(H)C=O fragment is at −2.5 eV but is significantly lowered for (H)C=S (−3.6 eV) and ⋅(H)C=Se (−3.8 eV) (see below). Therefore, more electron density is donated from the N_LP_ orbital at approximately −7 eV, into the lower‐lying π*_C=S_ and π*_C=Se_, compared to the high‐energy π*_C=O_ level. This larger flow of electronic density is reflected by the larger gross Mulliken populations observed for π*_C=S_ and π*_C=Se_ and by the larger contribution of the π*_C=X_ to the bonding π_1_ MO for S (11 %) and Se (13 %), compared to O (5 %) (both highlighted in yellow in Figure 7a). The orbital overlap of the π*_C=X_ with N_LP_ changes only minorly but decreases from O (0.29) to the heavier chalcogens S (0.26) and Se (0.25) following from the decreasing coefficient on carbon from π*_C=O_ to π*_C=S_ to π*_C=Se_ (Figure 7b). The more pronounced electron donation of the nitrogen lone pair into the π*_C=S_ and π*_C=Se_ orbitals is also responsible for the higher degree of planarization of the amino groups in thio‐ and selenourea (see above). The increase of charge donation of N_LP_ into the lower‐lying π*_C=S_ and π*_C=Se_ orbitals leads to shortening of the C−N bond going from **Ur**−**O** to **Ur**−**S** to **Ur**−**Se** (see Figure S5). Shortening of the C−N distance increases the steric repulsion between C and the H atoms attached to N, which favors a planar geometry of the NH_2_ groups that relieves the steric repulsion.^[17]^

### The nature of the C=X π*orbital

**Figure 8:**
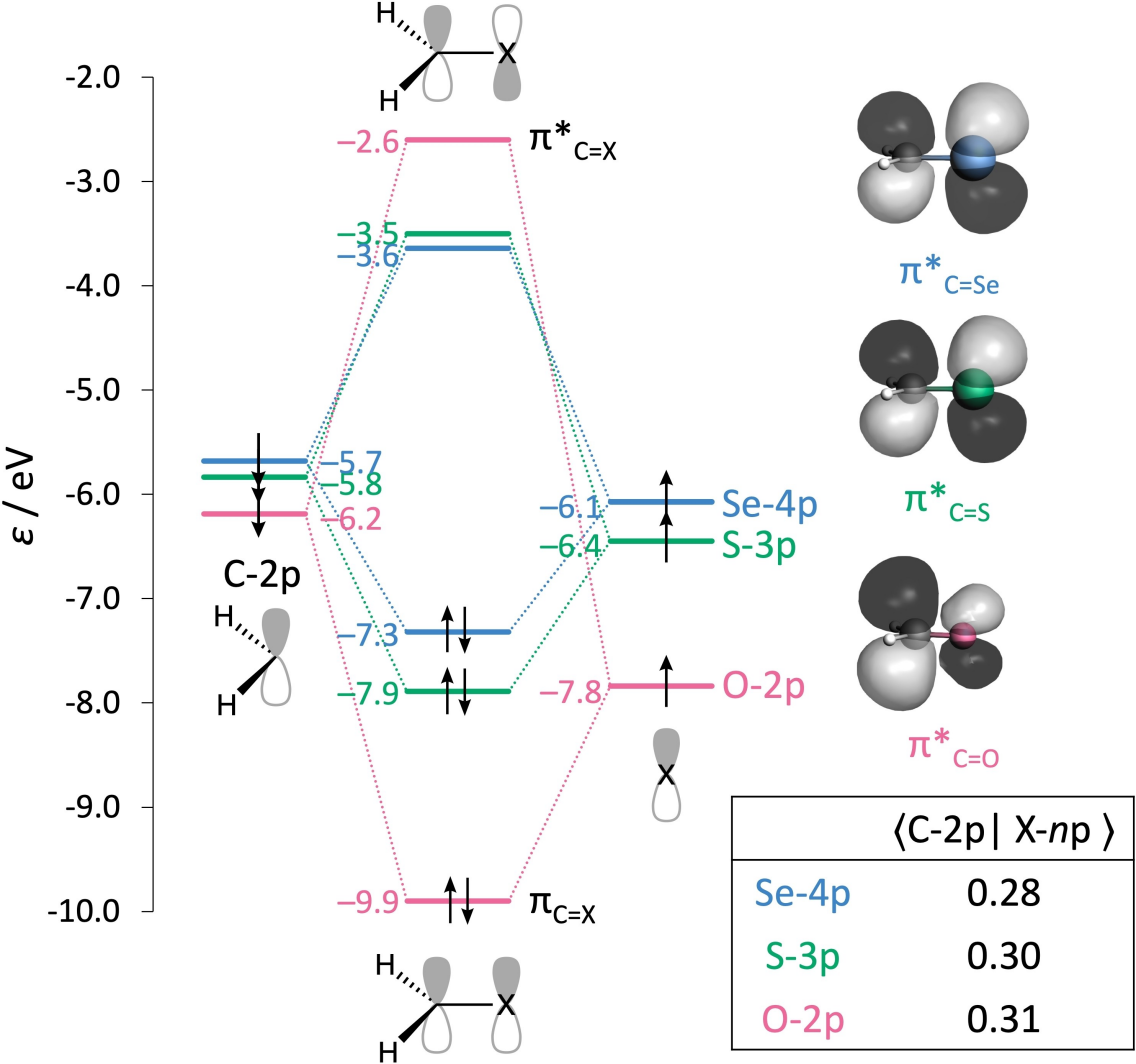
Orbital interaction diagram for the C=X π electron‐pair bond in **Al**−**X** with X=O, S, and Se, including orbital energies (in eV), isosurfaces (at 0.03 au) of the π*_C=X_ orbitals, and the orbital overlap between X‐*n*p and C‐2p.

Now, that we showed that the amino groups of thioamides and selenoamides are more positive than for carboxamides and that this is caused by the donation of electronic charge from N_LP_ of the amino group to their lower‐lying π*_C=X_ levels, we want to understand why the π*_C=X_ is lower for X=S and Se than for X=O. To this end, we turn to the most fundamental compounds containing a C=X double bond: aldehydes (**Al**−**X**). In Figure 8, we examine the π electron‐pair bond formation between a ⋅⋅CH_2_ fragment and the chalcogen atoms (⋅⋅X) in **Al**−**X**, where the singly occupied molecular orbital (SOMO) on carbon, which is a 2p atomic orbital (AO), overlaps laterally with the *n*p SOMO of the chalcogen atom, which is a 2p, 3p, and 4p AO for O, S, and Se, respectively (see Table S8 for gross Mulliken populations and MO contributions). The mixing of these p‐AOs results in a bonding (π_C=X_) and antibonding (π*_C=X_) π molecular orbital, where the latter is of similar nature as the π*_C=X_ orbital of the amides (see above). For the analogous orbital analysis of the C=X π electron‐pair bond in **Am**−**X**, see Figure S6. Note that the σ bond formation is not responsible for the positive charge accumulation and thus not considered.

The chalcogen *n*p SOMO is raised in energy descending Group 16 of the Periodic Table from −7.8 (O‐2p) to −6.4 (S‐3p) to −6.1 (Se‐4p) eV (see Figure 8). The destabilization of the *n*p level going from O to S to Se can be explained by the increase of the quantum number of the *n*p orbital along this trend. The electrons in the 3p and 4p orbitals of S and Se are simply further away from the nucleus than the electrons in the O‐2p orbital, and are therefore experiencing less nuclear attraction and are thus higher in energy.^[18]^ When the unpaired electrons in the two p‐AOs are combined upon forming the C=X π electron‐pair bond, they are stabilized in the bonding π_C=X_ molecular orbital accompanied by the generation of an empty antibonding π*_C=X_ orbital. The largest stabilization of the electron‐pair in the π_C=X_ bond is also associated with the largest destabilization of the antibonding π*_C=X_ level. Figure 8 shows that the stabilization of π_C=X_ is the largest for X=O and decreases for X=S and Se, so that the π*_C=O_ orbital is also the most destabilized, ending up the highest in energy, followed by the lower energy π*_C=S_ and π*_C=Se_, respectively.

**Figure 9:**
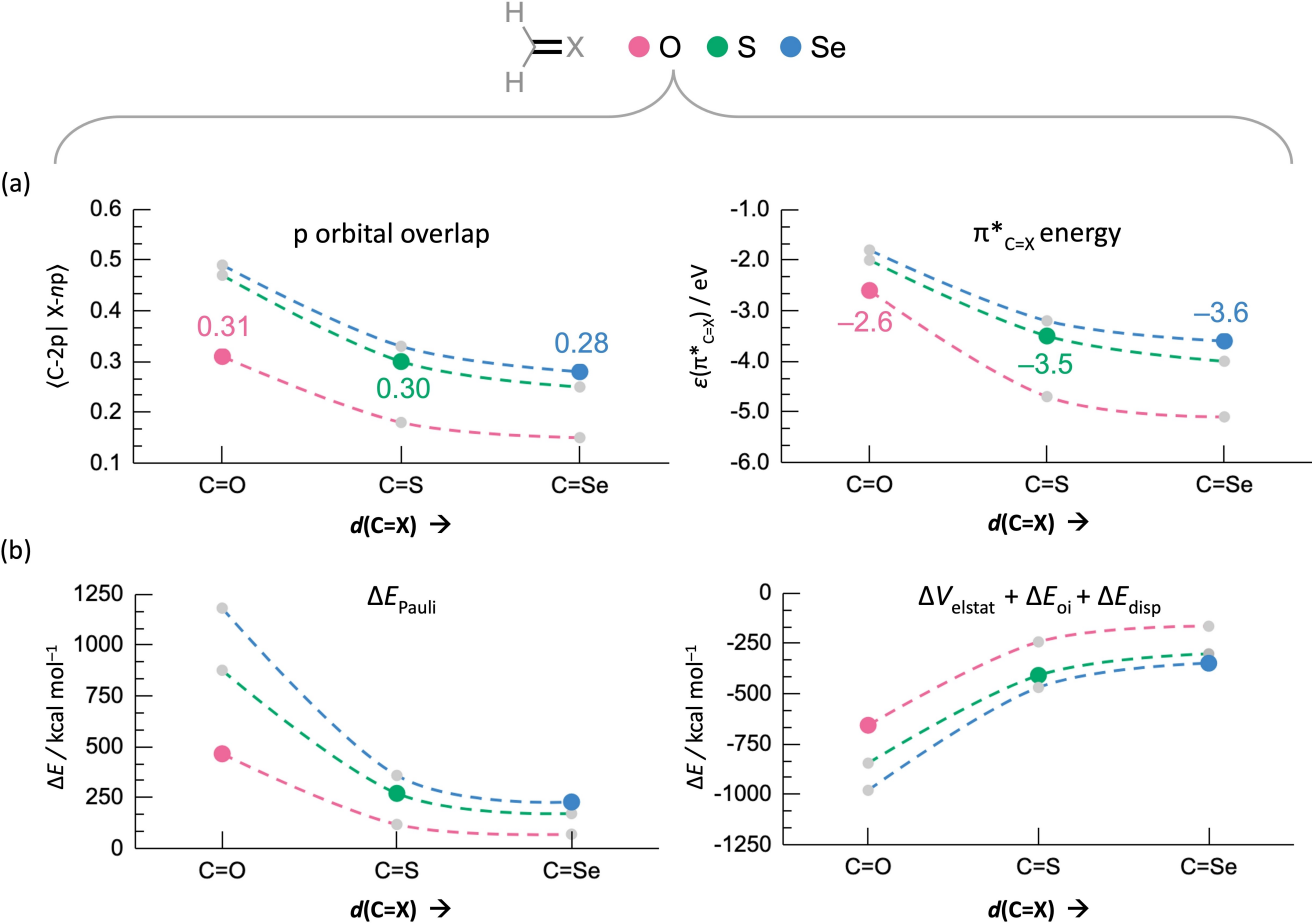
a) p orbital overlap (C‐2p|X‐*n*p), π*_C=X_ energy *ϵ* (in eV), b) the Pauli repulsion Δ*E*_Pauli_ (in kcal mol^−1^), and the sum of the stabilizing interactions (Δ*V*_elstat_+Δ*E*_oi_+Δ*E*_disp_), of the ⋅⋅CH_2_ fragment interacting with chalcogen atoms (⋅⋅X) in **Al**−**X** (with X=O, S, and Se) at the equilibrium *d*(C=X) of O (C=O), S (C=S), and Se (C=Se). All other bond distances and angles are frozen. The lines between the data points are there to guide the reader.

The stabilization of the electron‐pair π bond and destabilization of the π* orbital relative to the *n*p level is determined by the mutual overlap between the C‐2p and X‐*n*p SOMOs, and not by the relative energies of these orbitals (see Figure 8).[^19^, ^20^] The overlap between the SOMOs is the largest in the case of oxygen (0.31), followed by sulfur (0.30) and selenium (0.28), leading to the largest destabilization of the π*_C=X_ for oxygen. The decreasing SOMO‐SOMO overlap for the heavier chalcogens can be traced back to the increasing equilibrium C=X bond distance going from X=O (1.211 Å) to S (1.621 Å) to Se (1.771 Å). We prove that the relative energies of the π*_C=X_ levels indeed scale with the p‐orbital overlap by artificially adjusting the C=X bond distance (Figure 9). If the C=X distance is artificially put at the same distance for **Al**−**O**, **Al**−**S**, and **Al**−**Se** (see Figure 9a), we see that at the shortest distance, that is at the C=O equilibrium distance, selenium has the largest overlap and consequently the highest π*_C=X_ level.

**Figure 10:**
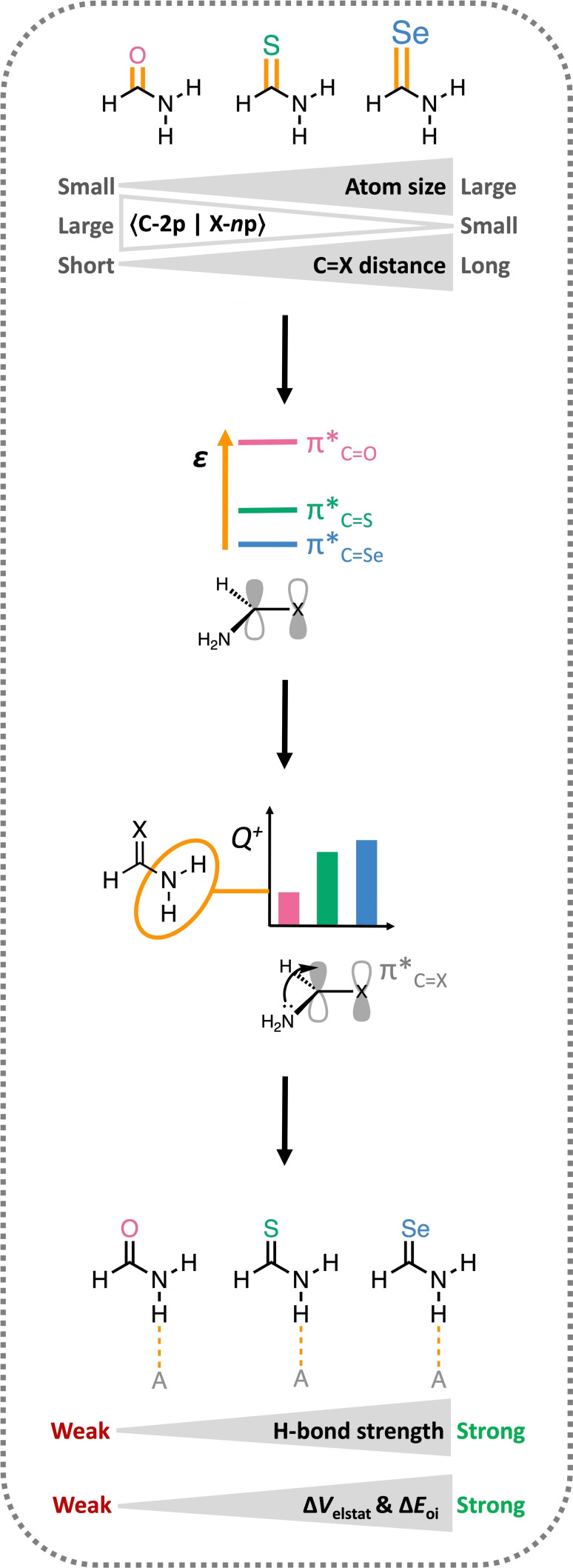
Schematic summary of the origin of the enhanced hydrogen‐bond donor strengths of thioamides (X=S) and selenoamides (X=Se) compared to carboxamides (X=O). “A” denotes a generic hydrogen‐bond acceptor.

Nevertheless, S and Se give rise to longer equilibrium C=X distances than O. The reason why the larger chalcogens cannot have short equilibrium C=X bonds is determined by their steric size, as is disclosed in Figure 9b. In this figure, the destabilizing steric Pauli repulsion Δ*E*_Pauli_ and the sum of the stabilizing interaction terms (Δ*V*_elstat_+Δ*E*_oi_+Δ*E*_disp_) are plotted as a function of the C=X distance (*d*(C=X)) for the interaction between ⋅⋅CH_2_ and ⋅⋅X in **Al**−**X** (see Figure S7 and Table S9 for the decomposition of all terms). (Note that the energy decomposition terms in Figure 9b are much larger than the energy terms of Figure 3, as Figure 9b involves the formation of strong covalent C=X bonds, while Figure 3 relates to rather weak intermolecular hydrogen‐bond interactions.) Although in Figure 9b the stabilizing energy terms become more stabilizing with about the same pace at shorter *d*(C=X) for all three chalcogens (i. e., roughly the same slope), the destabilizing Δ*E*_Pauli_ increases faster for the larger chalcogens S and Se (i. e., steeper slope) associated with the diffuseness and the larger number of core electrons of the 3^rd^ and 4^th^ period elements. As the 2^nd^ period element oxygen has the least increasing Δ*E*_Pauli_ at a shorter C=X distance, O can approach the C atom much closer resulting in the shortest equilibrium C=X distance, while the heavier chalcogens prefer longer C=X distances that diminish the Pauli repulsion. Therefore, the steric Pauli repulsion controls the equilibrium C=X distance, which determines the degree of p orbital overlap and consequently the energy of the π*_C=X_ level (Figure 9a). The amide hydrogen‐bond donor strength, which follows from the energy of this π*_C=X_ level (see above), is therefore determined by the effective steric size of the chalcogen atom X, and not by the electronegativity difference between X and C nor, as often suggested, the chalcogen's polarizability (see the summary in Figure 10).

We envisage that the findings in this work will contribute to the development of novel and improved amide‐based organocatalysts and supramolecular materials, as for rational design it is essential to fully understand the intrinsic hydrogen‐bond donor capability of amides. In this work, we show that the hydrogen‐bond donor strength of carboxamides, thioamides, and selenoamides is determined by the degree of electronic density flow from the NH_2_ groups to the π*_C=X_ orbital. From there on, one can tune the amide hydrogen‐bond donor strength by variation of the chalcogen atom or by introducing substituents that tune the energetic level of the π*_C=X_ orbital to arrive at a desired catalytic activity or polymer stability.

## Conclusion

Our computational analyses have elucidated why thioamides and selenoamides, widely used in organocatalysis and supramolecular chemistry, are better hydrogen‐bond donors than carboxamides. As the electronegativity difference between the chalcogens would have predicted the reverse order of hydrogen‐bond donor strengths, most studies use explanations in terms of the higher polarizability and charge capacity of heavier chalcogens. In this work, we demonstrate that neither the electronegativity nor the polarizability but the effective *steric size* of the chalcogen atom determines the hydrogen‐bond donor capability of the corresponding amide. This emerges from our dispersion‐corrected relativistic DFT computations within the framework of Kohn‐Sham molecular orbital theory.

The effective atomic size of the chalcogen atom increases from O to S to Se, and therefore, the amide C=X bond is elongated due to the increase of steric Pauli repulsion for the heavier, more diffuse, and electron‐rich chalcogens. Consequently, the S‐3p and Se‐4p atomic orbitals have a smaller overlap with the 2p atomic orbital of C than the O‐2p atomic orbital. This results in a weaker π interaction for S and Se and thus lowering of the π*_C=S_ and π*_C=Se_ levels compared to π*_C=O_. These energetically lower‐lying π*_C=S_ and π*_C=Se_ orbitals can accept more electron density from the nitrogen lone pair of the NH_2_ group than the high‐energy π*_C=O_ upon formation of the amide C−N bond. This larger flow of electrons leads to an increase of positive charge on the NH_2_ groups for thio‐ and selenoamides compared to carboxamides. The more positive NH_2_ groups give rise to enhanced orbital and electrostatic interactions upon hydrogen bonding. Thus, thioamides and selenoamides can form stronger hydrogen bonds than carboxamides and are therefore better hydrogen‐bond donor organocatalysts.

## Conflict of interest

The authors declare no conflict of interest.

1

## Supporting information

As a service to our authors and readers, this journal provides supporting information supplied by the authors. Such materials are peer reviewed and may be re‐organized for online delivery, but are not copy‐edited or typeset. Technical support issues arising from supporting information (other than missing files) should be addressed to the authors.

Supporting Information

## Data Availability

The data that support the findings of this study are available in the supplementary material of this article.
