# Supplementary material for: How the Chalcogen Atom Size Dictates the Hydrogen‐Bond Donor Capability of Carboxamides, Thioamides, and Selenoamides
Source: Chemistry. 2022 Apr 26;28(31):e202200755. doi: 10.1002/chem.202200755 (PMC9324920; doi:10.1002/chem.202200755)
Supplement: Supplementary file 1 — Supporting Information [file CHEM-28-0-s001.pdf]

Chemistry–A European Journal

Supporting Information

How the Chalcogen Atom Size Dictates the Hydrogen-Bond Donor Capability of Carboxamides, Thioamides, and Selenoamides

Celine Nieuwland and Célia Fonseca Guerra*

Contents

Method S1. Computational details 3

Method S2. Activation Strain Model (ASM) and Energy Decomposition Analysis (EDA) 5

Method S3. Voronoi deformation density analysis 7

Method S4. Fock matrix elements in the basis of fragment molecular orbitals 8

Discussion S1. Hydrogen-bond energy as function of the intermolecular distance 9

Figure S1. Decomposition of the interaction energy ΔE_{int} (in kcal mol^{-1}) of the hydrogen-bond interaction in the **F···Ur–X** complexes with X = O (pink), S (green), or Se (blue), as function of the hydrogen-bond distance O···(H)N (in Å), computed at ZORA-BLYP-D3(BJ)/TZ2P. The equilibrium O···(H)N distances are indicated by the dots (•) in the graph. The O···(H)N distance was varied in a stepwise manner (step size of 0.05 Å), while the molecular fragments were either frozen in the planar C_{2v} equilibrium geometry (constant) or were allowed to fully relax without symmetry constraints at each step (variational). 10

Figure S2. The electrostatic interaction ΔV_{elstat} (in kcal mol^{-1}) of the hydrogen-bond interaction in the **F···Ur–X** complexes, with X = O (pink), S (green), or Se (blue), at a hydrogen-bond distance O···(H)N of 3.25, 3.50, and 4.00 Å, computed at ZORA-BLYP-D3(BJ)/TZ2P while the geometries of the monomers were frozen in the planar C_{2v} equilibrium geometry. The lines between the data points are there to guide the reader. 11

Figure S3. Equilibrium hydrogen-bond (O···(H)N), C=X, and C–N distances (in Å) for the **F···Ur–X** and **F···Am–X** C_1 (non-planar) equilibrium complexes with X = O, S, and Se, optimized at ZORA-BLYP-D3(BJ)/TZ2P. Hydrogen-bond energies ΔE_{bond} (in kcal mol^{-1}) are shown below the structures between brackets. Color code of the ball-and-stick structures: hydrogen – white; carbon – grey; nitrogen – dark blue; oxygen – pink; sulfur – green; selenium – light blue. 12

Figure S4. Decomposition of the hydrogen-bond energy ΔE_{bond} (in kcal mol^{-1}) of the hydrogen-bond interaction in the **F···Ur–X** (left) and **F···Am–X** (right) C_1 (non-planar) equilibrium complexes with X = O, S, and Se, computed at ZORA-BLYP-D3(BJ)/TZ2P. 12

Figure S5. Equilibrium C–N distances (in Å) in the **Ur–X** and **Am–X** equilibrium geometries with X = O, S, and Se, optimized at ZORA-BLYP-D3(BJ)/TZ2P. For **Ur–X**, the sum of angles (in °) of the NH_2 group is indicated in red to show the planarization of the NH_2 group from X = O to Se. The **Am–X** monomers are planar. Color code of the ball-and-stick structures: hydrogen – white; carbon – grey; nitrogen – dark blue; oxygen – pink; sulfur – green; selenium – light blue. 13

Figure S6. Orbital interaction diagram for the C=X π electron-pair bond in the **Am–X** equilibrium geometries with X = O, S, and Se, including orbital energies (in eV) and the orbital overlap between X-*n*p and $\pi^*_{C\text{–}N}$. Computed at ZORA-BLYP-D3(BJ)/TZ2P. The FMO energies are calculated within the final molecular density (see Method S4). 13

Figure S7. Decomposition of the interaction energy ΔE_{int} (in kcal mol^{-1}) between the ··CH_2 fragment and the chalcogen atom (··X) in **Al–X** with X = O, S, and Se, at a C=X distance of 1.211, 1.621, and 1.771 Å, which is the equilibrium *d*(C=X) of **Al–O**, **Al–S** and **Al–Se**, respectively. All other bond distances and angles were frozen in the equilibrium geometry of the respecting **Al–X**. All energies are computed using an unrestricted formalism at ZORA-BLYP-D3(BJ)/TZ2P. The lines between the data points are there to guide the reader. 14

Table S1. Performance of various computational methods in the computation of the hydrogen-bond energy ΔE_{bond} (in kcal mol^{-1}) of the **F···Ur–X** and **F···Am–X** non-planar and planar complexes optimized at ZORA-BLYP-D3(BJ)/TZ2P. 15

Table S2. Decomposition of the bond energy ΔE_{bond} (in kcal mol^{-1}) of the hydrogen-bond interaction in the **F···Ur–X** and **F···Am–X** C_1 (non-planar) equilibrium complexes with X = O, S, and Se. **16**

Table S3. Decomposition of the bond energy ΔE_{bond} (in kcal mol^{-1}) of the hydrogen-bond interaction in the **F···Ur–X** and **F···Am–X** planar complexes with X = O, S, and Se. **17**

Table S4. Gross Mulliken populations (in electrons) and orbital overlap (S) of the molecular orbitals (MOs) involved in the hydrogen-bond interaction in the **F···Ur–X** and **F···Am–X** planar complexes with X = O, S, and Se. **18**

Table S5. Orbital energies ε (in eV) of the molecular orbitals (MOs) resulting from the hydrogen-bond interaction in the **F···Ur–X** and **F···Am–X** planar complexes with X = O, S, and Se. **18**

Table S6. Orbital energies ε (in eV) and orbital overlap of fragment molecular orbitals (FMOs), and the molecular orbitals (MOs) resulting from the π interaction upon C–N bond formation in **Am–X** (with X = O, S, and Se). **19**

Table S7. Decomposition of the interaction energy ΔE_{int} (in kcal mol^{-1}) upon C–N bond formation in **Am–X** (with X = O, S, and Se). **19**

Table S8. Gross Mulliken populations (in electrons), contributions (in %) to the $\pi_{C=X}$ and $\pi^*_{C=X}$ MOs, and orbital overlap of the p atomic orbitals involved in the π C=X bond formation in **Al–X** (with X = O, S, and Se). **19**

Table S9. Decomposition of the interaction energy ΔE_{int} (in kcal mol^{-1}), p orbital overlap, and the energy of the C=X π* level (in eV), of the ••CH_2 fragment interacting with the chalcogen atoms (••X) in **Al–X** at the equilibrium *d*(C=X) of **Al–O**, **Al–S**, and **Al–Se**. **20**

Table S10. Decomposition of the interaction energy ΔE_{int} (in kcal mol^{-1}) of the hydrogen-bond interaction in **F···Ur–X** (with X = O, S, and Se) as function of the hydrogen-bond distance O···(H)N (in Å). **20**

Table S11. List with Cartesian coordinates (in Å), total bond energies *E* (in kcal mol^{-1}), and number of imaginary frequencies (N_{imag}) of all geometries optimized at ZORA-BLYP-D3(BJ)/TZ2P. It is specified if no symmetry (C_1) or planar symmetry (C_s or C_{2v}) was enforced during the optimization process. **22**

Method S1. Computational details

All calculations were performed using the Amsterdam Density Functional (ADF) program (ADF2019.102 and for the unrestricted analyses (Figure 7-9 in the main text) version ADF2019.302).[1] Geometry optimizations and single-point energy calculations were based on dispersion-corrected relativistic density functional theory (DFT-D) at the ZORA-BLYP-D3(BJ) level of theory in the gas phase.[2] A TZ2P basis set was used in combination with a small frozen core for the geometry optimizations, and no frozen core was used for the molecular orbital analysis.[1a-c] The TZ2P basis set consists of an uncontracted set of Slater-type orbitals (STOs), which is of triple-ζ quality for all atoms and has been augmented with two sets of polarization functions. The quality of the density fitting (ZlmFit) and the integration grid (BeckeGrid) was set to "verygood" for all computations.[3]

In addition, single point energies were computed at ZORA-BLYP-D3(BJ)/QZ4P and ZORA-M06-2X[4]/TZ2P on fully optimized ZORA-BLYP-D3(BJ)/TZ2P geometries to identify the effect of the basis set super position error (BSSE) and of meta-hybrid exchange-correlation functionals on the computed hydrogen-bond energy trends, respectively. Furthermore, the hydrogen-bond energies were computed using Domain-based Local Pair Natural Orbital Coupled Cluster (DLPNO-CCSD(T))[5] calculations using the ORCA[5a] 5.0.0 program with TightPNO and the def2-QZVPP[6] basis set on ZORA-BLYP-D3(BJ)/TZ2P geometries. The data of this performance analysis, presented in Tables S1-S3, shows the same trends in hydrogen-bond energies and decomposition terms for the investigated systems and displays that all-ZORA-BLYP-D3(BJ)/TZ2P, ZORA-BLYP-D3(BJ)/QZ4P//ZORA-BLYP-D3(BJ)/TZ2P, and ZORA-M06-2X/TZ2P//ZORA-BLYP-D3(BJ)/TZ2P perform equally well.

[1] a) G. te Velde, F. M. Bickelhaupt, E. J. Baerends, C. Fonseca Guerra, S. J. A. van Gisbergen, J. G. Snijders, T. Ziegler, *J. Comput. Chem*. **2001**, *22*, 931–967; b) E. J. Baerends, D. E. Ellis, P. Ros, *Chem. Phys.* **1973**, *2*, 41–51; c) E. van Lenthe, E. J. Baerends, *J. Comput. Chem.* **2003**, *24*, 1142–1156; d) C. Fonseca Guerra, J. G. Snijders, G. te Velde, E. J. Baerends, *Theor. Chem. Acc*. **1998**, *99*, 391–403; e) ADF2019.102, SCM Theoretical Chemistry; Vrije Universiteit, Amsterdam, The Netherlands, www.scm.com.

[2] a) A. D. Becke, *Phys. Rev. A* **1988**, *38*, 3098–3099; b) C. Lee, W. Yang, R. G. Parr, *Phys. Rev. B* **1988**, *37*, 785–789; c) Q. Wu, W. Yang, *J. Chem. Phys*. **2002**, *116*, 515–524; d) S. Grimme, *J. Comput. Chem.* **2004**, *25*, 1463–1473; e) S. Grimme, *J. Comput. Chem.* **2006**, *27*, 1787–1799; f) S. Grimme, J. Antony, S. Ehrlich, H. Krieg, *J. Chem. Phys.* **2010**, *132*, 154104; g) S. Grimme, S. Ehrlich, L. Goerigk, *J. Comput. Chem.* **2011**, *32*, 1456–1465; h) E. van Lenthe, A. Ehlers, E. J. Baerends, *J. Chem. Phys.* **1999**, *110*, 8943–8953.

[3] a) M. Franchini, P. H. T. Philipsen, E. van Lenthe, L. Visscher, *J. Chem. Theory Comput.* **2014**, *10*, 1994–2004; b) M. Franchini, P. H. T. Philipsen, L. Visscher, *J. Comput. Chem.* **2013**, *34*, 1819–1827.

[4] a) Y. Zhao, D. G. Truhlar, *J. Chem. Phys.* **2006**, *125*, 194101; b) Y. Zhao, D. G. Truhlar, *Theor. Chem. Acc.* **2008**, *120*, 215–241.

[5] a) F. Neese, F. Wennmohs, U. Becker, C. Riplinger, *J. Chem. Phys.* 2020, **152**, 224108; b) C. Riplinger, B. Sandhoefer, A. Hansen, F. Neese, *J. Chem. Phys.* **2013**, *139*, 134101.

[6] a) F. Weigend, R. Ahlrichs, *Phys. Chem. Chem. Phys*. **2005**, *7*, 3297–3305; b) F. Weigend, *Phys. Chem. Chem. Phys.* **2006**, *8*, 1057–1065.

All optimized geometries have been checked to be energy minima by performing a vibrational frequency analysis (*i.e.,* no imaginary frequencies for minima, see Table S11).[7]

Fragment molecular orbital (FMO) energies of molecular fragments that form electron-pair bonds in the interacting complex are computed within the electronic density of the final molecule (see Method S4 for a detailed explanation of this approach). The molecular structures in this work are illustrated with CYLview20.[8]

[7] a) S. K. Wolff, *Int. J. Quantum Chem.* **2005**, *104*, 645–659; b) A. Bérces, R. M. Dickson, L. Fan, H. Jacobsen, D. P. Swerhone, T. Ziegler, *Comput. Phys. Commun.* **1997**, *100*, 247–262; c) H. Jacobsen, A. Bérces, D. P. Swerhone, T. Ziegler, *Comput. Phys. Commun.* **1997**, *100*, 263–276.

[8] C. Y. Legault, *CYLview20*, Université de Sherbrooke, Sherbrooke, Quebec, Canada, **2020**, www.cylview.org.

Method S2. Activation Strain Model (ASM) and Energy Decomposition Analysis (EDA)

In this work two types of hydrogen-bonded systems are studied: formaldehyde–chalcourea (**F···Ur–X**, with X = O, S, and Se) and formaldehyde–chalcoamide (**F···Am–X**). **F···Ur–X** are complexes containing two hydrogen bonds, the so-called bifurcated hydrogen bonds, whereas the **F···Am–X** complexes comprise a single hydrogen bond. The hydrogen-bond energy (ΔE_{bond}) between **Ur–X** or **Am–X** and **F** is formulated by Equation S1a and S1b, respectively.

$$\Delta E_{\text{bond}} = E(\mathbf{F\cdots Ur–X}) – E(\mathbf{Ur–X}) – E(\mathbf{F}) \qquad \text{(S1a)}$$
$$\Delta E_{\text{bond}} = E(\mathbf{F\cdots Am–X}) – E(\mathbf{Am–X}) – E(\mathbf{F}) \qquad \text{(S1b)}$$

In these equations E(**Ur–X**), E(**Am–X**), and E(**F**) correspond to the energies of the chalcourea, chalcoamide, and formaldehyde molecules in their equilibrium geometry, respectively. E(**F···Ur–X**) and E(**F···Am–X**) denote the energies of the corresponding optimized hydrogen-bonded complexes (non-planar (C_1) or enforced planar (C_s or C_{2v})).

For the **F···Am–X** complexes a C_1 (non-planar) and a C_s symmetric (planar) minimum (*i.e.,* no imaginary frequencies) were found that are very close in energy (see Table S11 for the Cartesian coordinates and total bond energies of the optimized structures). For each of the **F···Ur–X** complexes one C_1 minimum was identified, but we obtained through a constrained optimization analogous C_{2v} symmetric structures that are close in energy and display only imaginary frequencies associated with the pyramidalization of the amino groups. In our analyses, we focus primarily on the C_{2v} **F···Ur–X** and C_s **F···Am–X** complexes, to which we refer as "planar complexes". These planar structures furnish identical hydrogen-bond energy trends as the non-planar C_1 complexes (see Tables S1-S3).

To understand the different components that determine the trends in ΔE_{bond}, the energy was partitioned as formulated by Equation S2 according to the activation strain model (ASM) of reactivity and bonding.[9]

$$\Delta E_{\text{bond}} = \Delta E_{\text{strain}} + \Delta E_{\text{int}} \qquad \text{(S2)}$$

Here, the strain energy (ΔE_{strain}) is the energy required to deform the equilibrium geometry of each fragment, to the geometry it acquires when it interacts in the hydrogen-bonded complex. The interaction energy (ΔE_{int}) accounts for the stabilizing interaction between the two deformed fragments.

[9] a) P. Vermeeren, S. C. C. van der Lubbe, C. Fonseca Guerra, F. M. Bickelhaupt, T. A. Hamlin, *Nat. Protoc.* **2020**, *15*, 649–667; b) I. Fernández, F. M. Bickelhaupt, *Chem. Soc. Rev.* **2014**, *43*, 4953–4967; c) F. M. Bickelhaupt, K. N. Houk, *Angew. Chem. Int. Ed.* **2017**, *56*, 10070–10086; *Angew. Chem.* **2017**, *129*, 10204–10221; d) P. Vermeeren, T. A. Hamlin, F. M. Bickelhaupt, *Chem. Commun.* **2021**, *57*, 5880–5896.

The interaction energy can be further decomposed based on the Kohn-Sham molecular orbital theory using a quantitative energy decomposition analysis (EDA), which divides the total interaction energy (ΔE_{int}) into Pauli repulsion (ΔE_{Pauli}), electrostatic interaction (ΔV_{elstat}), orbital interaction (ΔE_{oi}), and dispersion (ΔE_{disp}) energy components (see Equation S3).[10]

$$\Delta E_{int} = \Delta E_{Pauli} + \Delta V_{elstat} + \Delta E_{oi} + \Delta E_{disp} \quad \text{(S3)}$$

ΔE_{Pauli} comprises the destabilizing interactions arising from overlapping occupied orbitals and accounts for any steric repulsion. ΔV_{elstat} corresponds to classical electrostatic interactions between the unperturbed charge distributions of the prepared (*i.e.,* deformed) interacting molecular fragments and is usually attractive. The term ΔE_{oi} includes charge transfer (*i.e.,* donor–acceptor interactions between occupied orbitals on one of the interacting fragments and unoccupied orbitals on the other, including HOMO–LUMO interactions), polarization (empty–occupied orbital mixing on one fragment due to the presence of the other fragment), and electron-pair interactions (*e.g.,* SOMO–SOMO interactions). Finally, the ΔE_{disp} energy term includes a dispersion correction.

The orbital interaction term (ΔE_{oi}) can be further decomposed into the contributions from each irreducible representation Γ of the point group of the system regarded. For the calculations in this work planar structures are considered (C_s or C_{2v}), which allows for the decomposition of ΔE_{oi} into the σ and π orbital contributions (see Equation S4).

$$\Delta E_{oi} = \Delta E_{\sigma} + \Delta E_{\pi} \quad \text{(S4)}$$

[10] a) F. M. Bickelhaupt, E. J. Baerends, in *Reviews in Computational Chemistry*, Vol. 15 (Eds.: K. B. Lipkowitz, D. B. Boyd), Wiley-VCH, Weinheim, **2000**, pp. 1–86; b) T. A. Hamlin, P. Vermeeren, C. Fonseca Guerra, F. M. Bickelhaupt, in *Complementary Bonding Analyses* (Ed.: S. Grabowsky), De Gruyter, Berlin, **2021**, pp. 199–212.

Method S3. Voronoi deformation density analysis

The Voronoi deformation density (VDD) method allows for the analysis of the electronic redistributions within mono- or polyatomic fragments when a chemical bond is formed between these fragments.[11] The VDD atomic charge ($Q_{\mathrm{A}}^{\mathrm{VDD}}$) of atom A is given by Equation S5.

$$Q_{\mathrm{A}}^{\mathrm{VDD}} = -\int_{\text{Voronoi cell of A}} [\,\rho(\mathbf{r}) - \textstyle\sum_{\mathrm{B}} \rho_{\mathrm{B}}(\mathbf{r})\,]\,d\mathbf{r} \tag{S5}$$

Equation S5 uses numerical integration over the deformation density [$\rho(\mathbf{r}) - \sum_{\mathrm{B}} \rho_{\mathrm{B}}(\mathbf{r})$]: the density change going from a superposition of atomic densities, which is a fictitious promolecule, to the density in the final molecular system. This promolecule density is defined as the sum over the (spherically averaged) ground-state atomic densities $\sum_{\mathrm{B}} \rho_{\mathrm{B}}(\mathbf{r})$. The Voronoi cell of an atom is the space defined by the bond midplanes on and perpendicular to all bond axes between the nucleus and its neighboring nuclei.

The VDD analysis can also be used for studying the bonding between molecular fragments. Here the charge rearrangement ($\Delta Q_{\mathrm{A}}^{\mathrm{VDD}}$) compared to the initial density of the polyatomic fragments is measured and gives insight into the change in electronic density upon interacting (see Equation S6). $\Delta Q_{\mathrm{A}}^{\mathrm{VDD}}$ is an indication for the number of electrons that flows into ($\Delta Q_{\mathrm{A}}^{\mathrm{VDD}} < 0$) or out of ($\Delta Q_{\mathrm{A}}^{\mathrm{VDD}} > 0$) the Voronoi cell of a nucleus A as the result of the interaction between the two molecular fragments.

$$\Delta Q_{\mathrm{A}}^{\mathrm{VDD}} = -\int_{\text{Voronoi cell of A in molecule}} [\,\rho_{\text{molecule}}(\mathbf{r}) - \textstyle\sum_{\text{subsystems}} \rho_i(\mathbf{r})\,]\,d\mathbf{r} \tag{S6}$$

To analyze the charge shifts caused by charge transfer within the σ system or the π electron system, $\Delta Q_{\mathrm{A}}^{\mathrm{VDD}}$ can be further decomposed into the contributions of the σ ($\Delta Q_{\mathrm{A}}^{\sigma}$) and π ($\Delta Q_{\mathrm{A}}^{\pi}$) deformation density (see Equation S7). This approach is possible due to the use of C_{2v} or C_s symmetry in the computations.

$$\Delta Q_{\mathrm{A}}^{\Gamma} = -\int_{\text{Voronoi cell of A in molecule}} [\,\rho_{\text{molecule}}^{\Gamma}(\mathbf{r}) - \rho_{\text{Fragment1}}^{\Gamma}(\mathbf{r}) - \rho_{\text{Fragment2}}^{\Gamma}(\mathbf{r})\,]\,d\mathbf{r} \tag{S7}$$

The density ρ^{Γ} is the sum of the occupied molecular orbitals densities within a particular irreducible representation Γ. In this work, we show that the use of VDD analyses for different irreducible representations is a powerful tool for qualitative prediction of chemical bonding.

[11] a) C. Fonseca Guerra, J. W. Handgraaf, E. J. Baerends, F. M. Bickelhaupt, *J. Comput. Chem.* **2004**, *25*, 189–210; b) O. A. Stasyuk, H. Szatylowicz, T. M. Krygowski, C. Fonseca Guerra, *Phys. Chem. Chem. Phys.* **2016**, *18*, 11624–11633.

Method S4. Fock matrix elements in the basis of fragment molecular orbitals[12]

Within the ADF program there is the possibility to analyze the interaction between two molecular fragments by building up the molecule from mono- or polyatomic fragments. In this approach, the molecular orbitals $\{\psi_i^\Gamma\}$ that are computed are expressed as linear combinations of the symmetry-adapted fragment orbitals $\{\varphi_j^\Gamma\}$, which in turn can be written as linear combinations of STO basis functions $\{\chi_k\}$ (see Equation S8).

$$\psi_i^\Gamma = \sum_j a_{ij}^\Gamma \varphi_j^\Gamma = \sum_k b_{ik}^\Gamma \chi_k \qquad \text{(S8)}$$

For the Kohn-Sham Fock matrix ($\mathbf{F_{KS}}$) constructed from the symmetry-adapted fragment orbitals, the generalized Eigenvalue problem can be solved by giving the matrix eigenvectors $\mathbf{C}$ and corresponding eigenvalues in the diagonal matrix $\mathbf{E}$ (see Equation S9). In this equation $\mathbf{S}$ corresponds to the overlap matrix.

$$\mathbf{F_{KS}}\,\mathbf{C} = \mathbf{S}\,\mathbf{C}\,\mathbf{E} \qquad \text{(S9)}$$

The Kohn-Sham Fock matrix elements, given by Equation S10, correspond to the coupling or interaction strength between two orbitals φ_i^Γ and φ_j^Γ. The diagonal matrix elements denote the energies ε_i of the fragment orbitals (Equation S11), as a solution to the generalized Eigenvalue problem of Equation S9.

$$(F_{KS})_{ij} = \langle \varphi_i^\Gamma | \hat{F}_{KS} | \varphi_j^\Gamma \rangle \qquad \text{(S10)}$$

$$\varepsilon_i = \langle \psi_i^\Gamma | \hat{F}_{KS} | \psi_i^\Gamma \rangle \qquad \text{(S11)}$$

All matrix elements can be obtained either with the potential of the promolecular density (*i.e.,* the initial sum of fragment densities) or with the potential of the final molecular density. The former case corresponds to the field to which the orbitals are exposed before they start interacting but after the fragments have adopted the geometry in the final molecule. In the latter case, the fragment orbital energies are computed in the field of the final molecular density. In this work we calculate the fragment molecular orbital (FMO) energies of molecular fragments that form electron-pair bonds in the interacting complex within the electronic density of the final molecule (*e.g.,* Figures 7-8 in the main text).

---

[12] a) F. M. Bickelhaupt, M. Solà, C. Fonseca Guerra, *J. Mol. Model.* **2006**, *12*, 563–568; b) F. M. Bickelhaupt, M. Solà, C. Fonseca Guerra, *J. Comput. Chem.* **2006**, *28*, 238–250; c) F. M. Bickelhaupt, M. Solà, C. Fonseca Guerra, *Inorg. Chem.* **2007**, *46*, 5411–5418.

Discussion S1. Hydrogen-bond energy as function of the intermolecular distance

The energy decomposition analysis (EDA) terms were scanned as a function of the O···(H)N distance for the **F···Ur–X** complexes (Figure S1, see Table S10 for the numerical values) as this allows us to pinpoint which term is responsible for the interaction energy minimum and the equilibrium hydrogen-bond distance of the different complexes (see previous work in Ref. [13]). These EDA calculations were performed using the PyFrag 2019 program.[14] In this analysis the O···(H)N distance was varied in a stepwise manner (step size of 0.05 Å), while the molecular fragments were either frozen in their planar equilibrium geometries (constant) or were allowed to fully relax without symmetry constraints at each step (variational). As can be seen from Figure S1, the relative trends (O vs S vs Se) of the energy decomposition terms remain unchanged as function of the hydrogen-bond distance. More importantly, the constant and variational EDA method furnish the same trends which justifies the use of planar complexes in our analyses.

Although the trends of all energy terms in Figure S1 remain the same over the entire hydrogen-bond distance range, relevant information can be obtained from the slopes of the graph lines that show that ΔE_{oi} determines the equilibrium geometry. In Figure S1, the tendency for hydrogen bond contraction by ΔE_{disp} and $\Delta V_{\mathrm{elstat}}$, and elongation by the $\Delta E_{\mathrm{Pauli}}$ term, is roughly equal for all three chalcogens (*i.e.,* the graph lines are parallel). However, the ΔE_{oi} term decreases the fastest for Se as the hydrogen-bond distance increases (*i.e.,* steepest slope) in such a way that **Ur–Se** has the highest tendency for contraction of the hydrogen bond, while this tendency is less (*i.e.,* less steep slope) in the case of S and O. Because of this difference in ΔE_{oi}, the shortest hydrogen-bond distance is observed for **F···Ur–Se** and increases for the thioxo and carboxo complexes (see Figure 2 in main text and Figure S3). Although the slopes of the lines do not differ for $\Delta V_{\mathrm{elstat}}$ within the hydrogen-bond distance range of Figure S1, we observe that at longer intermolecular distance (3.25 Å ≤ [O···(H)N] ≤ 4.00 Å) the electrostatic interaction weakly contributes to the increasing tendency for hydrogen bond contraction for the heavier chalcogens (see Figure S2).

[13] a) S. C. C. van der Lubbe, F. Zaccaria, X. Sun, C. Fonseca Guerra, *J. Am. Chem. Soc.* **2019**, *141*, 4878–4885; b) S. C. C. van der Lubbe, C. Fonseca Guerra, *Chem. Eur. J.* **2017**, *23*, 10249–10253.

[14] a) X. Sun, T. M. Soini, J. Poater, T. A. Hamlin, F. M. Bickelhaupt, *J. Comp. Chem.* **2019**, *40*, 2227–2233; b) X. Sun, T. M. Soini, L. P. Wolters, W. J. van Zeist, C. Fonseca Guerra, T. A. Hamlin, F. M. Bickelhaupt, *PyFrag 2019*, Vrije Universiteit Amsterdam, Amsterdam, The Netherlands, **2019**, www.theochem.nl/pyfrag2019.

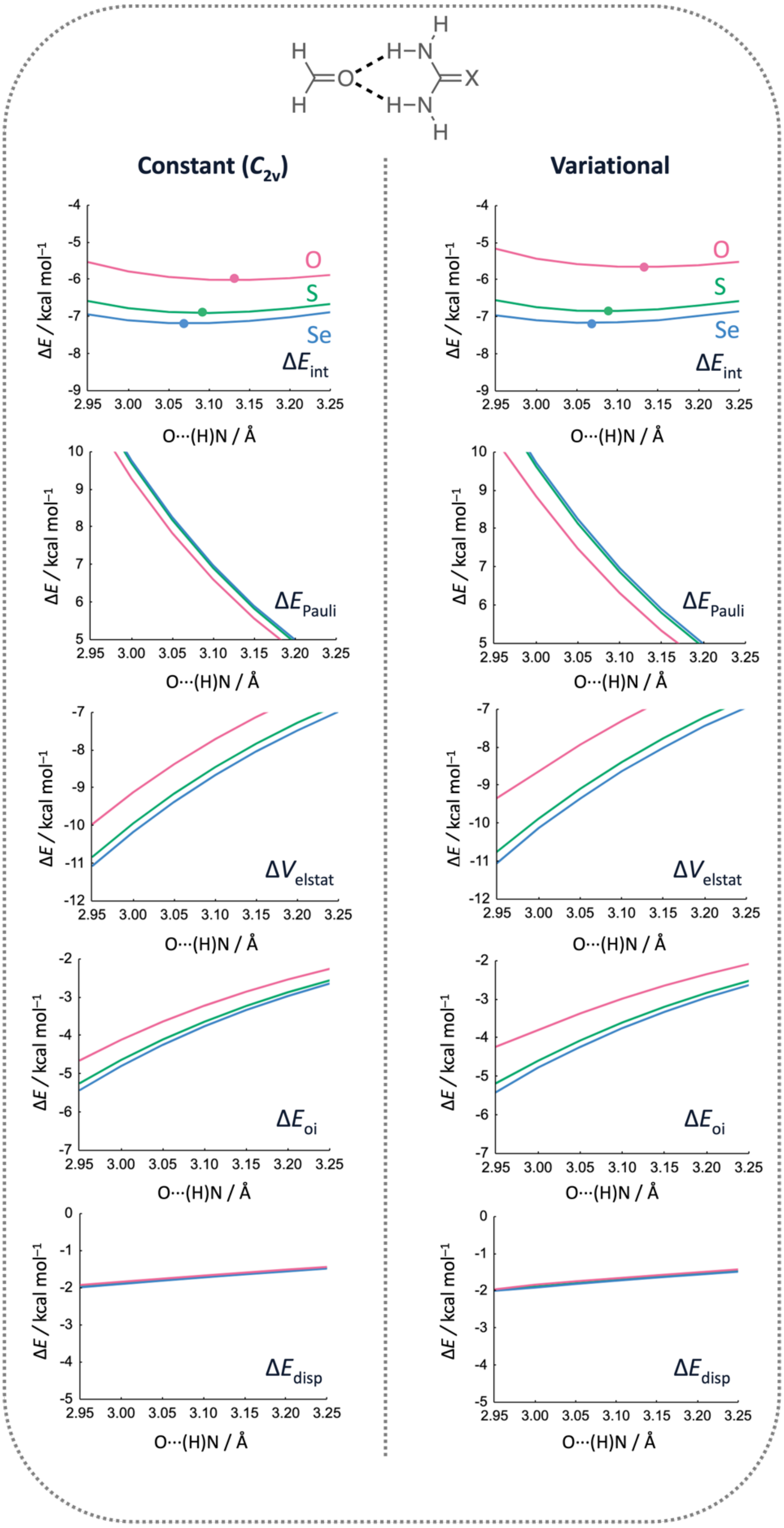

Figure S1. Decomposition of the interaction energy ΔE_{int} (in kcal mol^{-1}) of the hydrogen-bond interaction in the **F···Ur–X** complexes with X = O (pink), S (green), or Se (blue), as function of the hydrogen-bond distance O···(H)N (in Å), computed at ZORA-BLYP-D3(BJ)/TZ2P. The equilibrium O···(H)N distances are indicated by the dots (•) in the graph. The O···(H)N distance was varied in a stepwise manner (step size of 0.05 Å), while the molecular fragments were either frozen in the planar C_{2v} equilibrium geometry (constant) or were allowed to fully relax without symmetry constraints at each step (variational).

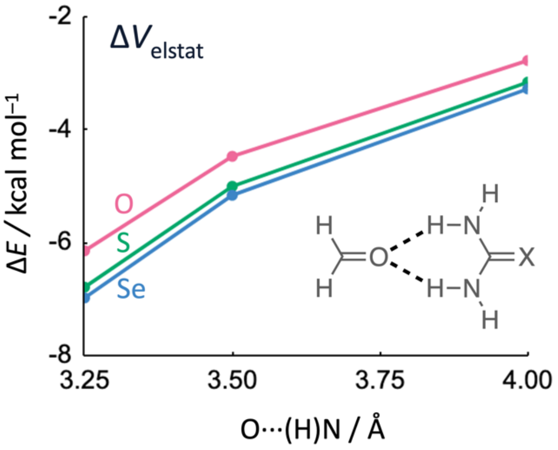

Figure S2. The electrostatic interaction ΔV_{elstat} (in kcal mol^{-1}) of the hydrogen-bond interaction in the **F···Ur–X** complexes, with X = O (pink), S (green), or Se (blue), at a hydrogen-bond distance O···(H)N of 3.25, 3.50, and 4.00 Å, computed at ZORA-BLYP-D3(BJ)/TZ2P while the geometries of the monomers were frozen in the planar C_{2v} equilibrium geometry. The lines between the data points are there to guide the reader.

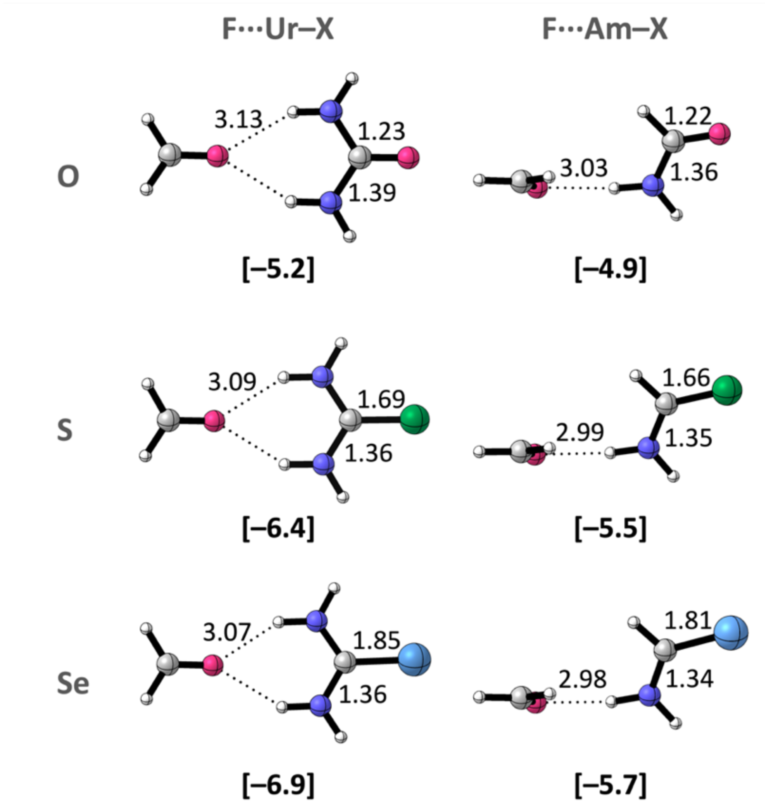

Figure S3. Equilibrium hydrogen-bond (O···(H)N), C=X, and C–N distances (in Å) for the **F···Ur–X** and **F···Am–X** C_1 (non-planar) equilibrium complexes with X = O, S, and Se, optimized at ZORA-BLYP-D3(BJ)/TZ2P. Hydrogen-bond energies ΔE_{bond} (in kcal mol^{-1}) are shown below the structures between brackets. Color code of the ball-and-stick structures: hydrogen – white; carbon – grey; nitrogen – dark blue; oxygen – pink; sulfur – green; selenium – light blue.

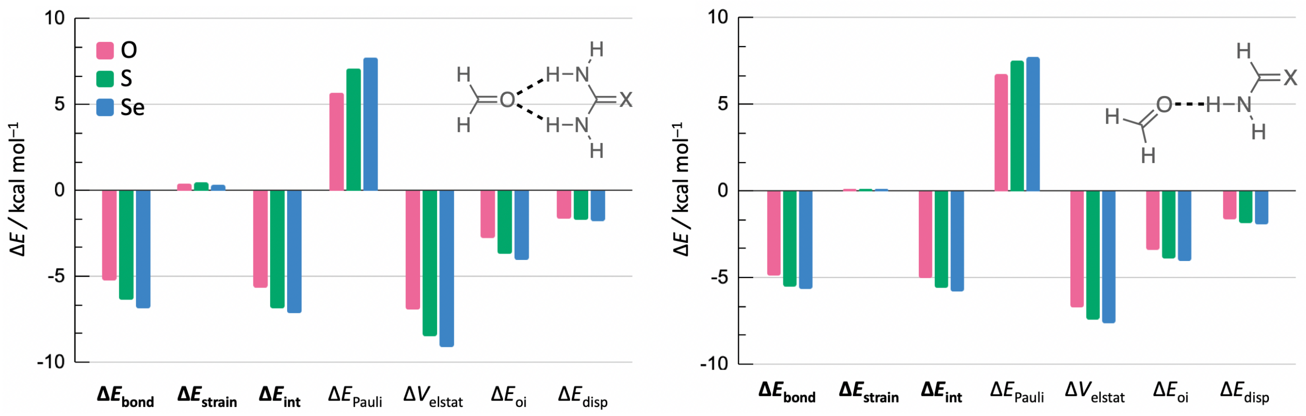

Figure S4. Decomposition of the hydrogen-bond energy ΔE_{bond} (in kcal mol^{-1}) of the hydrogen-bond interaction in the **F···Ur–X** (left) and **F···Am–X** (right) C_1 (non-planar) equilibrium complexes with X = O, S, and Se, computed at ZORA-BLYP-D3(BJ)/TZ2P.

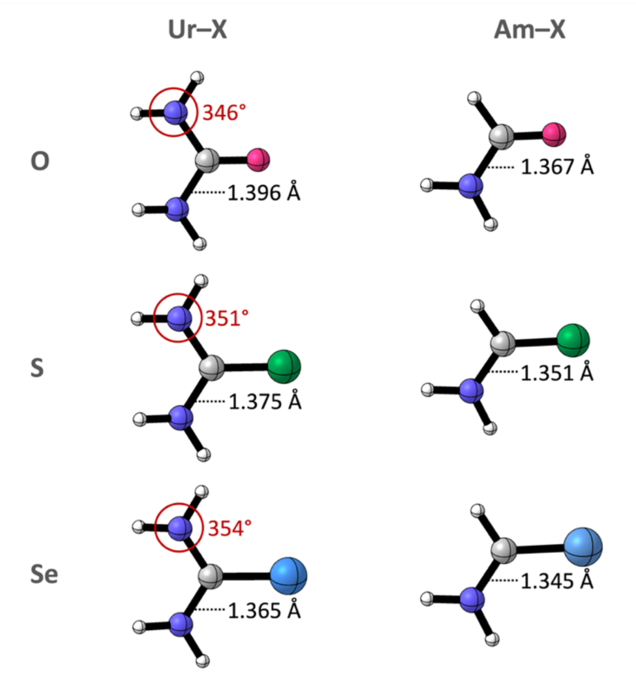

Figure S5. Equilibrium C–N distances (in Å) in the **Ur–X** and **Am–X** equilibrium geometries with X = O, S, and Se, optimized at ZORA-BLYP-D3(BJ)/TZ2P. For **Ur–X**, the sum of angles (in °) of the NH_2 group is indicated in red to show the planarization of the NH_2 group from X = O to Se. The **Am–X** monomers are planar. Color code of the ball-and-stick structures: hydrogen – white; carbon – grey; nitrogen – dark blue; oxygen – pink; sulfur – green; selenium – light blue.

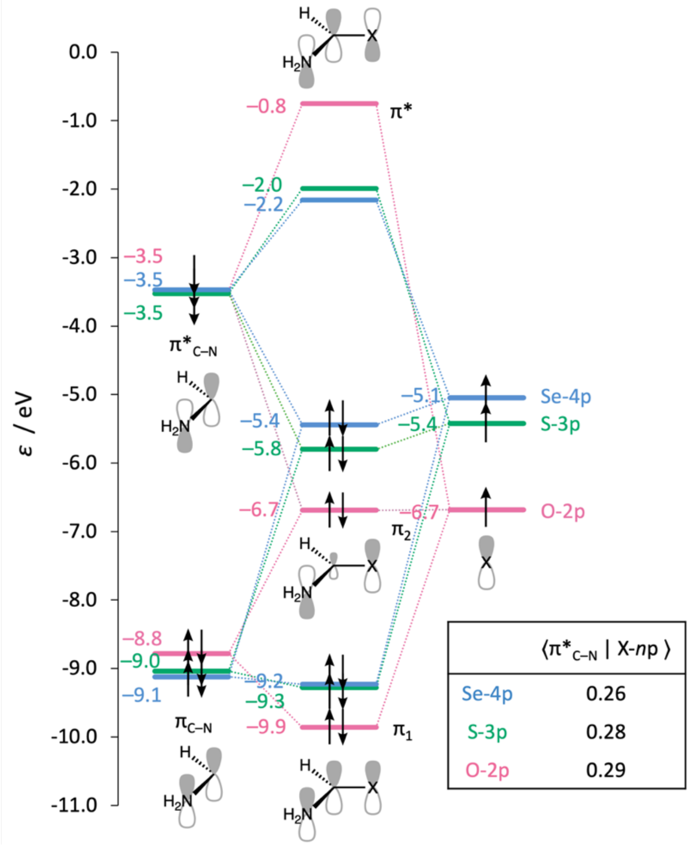

Figure S6. Orbital interaction diagram for the C=X π electron-pair bond in the **Am–X** equilibrium geometries with X = O, S, and Se, including orbital energies (in eV) and the orbital overlap between X-np and $\pi^*_{C\text{–}N}$. Computed at ZORA-BLYP-D3(BJ)/TZ2P. The FMO energies are calculated within the final molecular density (see Method S4).

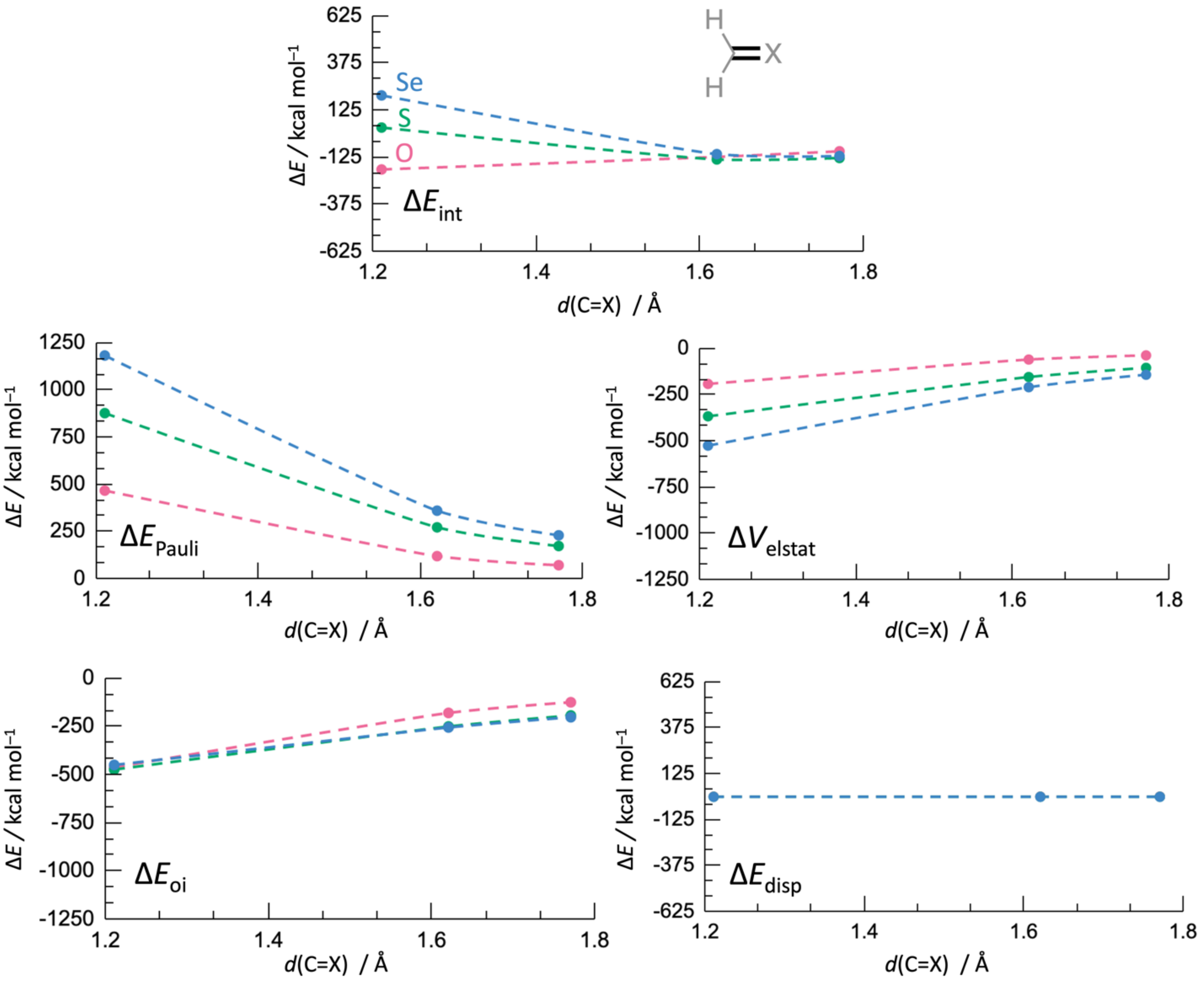

Figure S7. Decomposition of the interaction energy ΔE_{int} (in kcal mol^{-1}) between the ··CH_2 fragment and the chalcogen atom (··X) in **Al–X** with X = O, S, and Se, at a C=X distance of 1.211, 1.621, and 1.771 Å, which is the equilibrium *d*(C=X) of **Al–O**, **Al–S** and **Al–Se**, respectively. All other bond distances and angles were frozen in the equilibrium geometry of the respecting **Al–X**. All energies are computed using an unrestricted formalism at ZORA-BLYP-D3(BJ)/TZ2P. The lines between the data points are there to guide the reader.

Table S1. Performance of various computational methods in the computation of the hydrogen-bond energy ΔE_{bond}[a] (in kcal mol^{-1}) of the **F···Ur–X** and **F···Am–X** non-planar and planar[b] complexes optimized at ZORA-BLYP-D3(BJ)/TZ2P.

Non-planar (C_1)				
System	DLPNO-CCSD(T)[c]	ZORA-BLYP-D3(BJ)/TZ2P[d]	ZORA-BLYP-D3(BJ)/QZ4P	ZORA-M06-2X/TZ2P
**F···Ur–O**	–5.3	–5.2	–5.0	–5.5
**F···Ur–S**	–6.5	–6.4	–6.1	–6.9
**F···Ur–Se**	–7.1	–6.9	–6.6	–7.5
**F···Am–O**	–4.8	–4.9	–4.7	–4.7
**F···Am–S**	–5.4	–5.5	–5.2	–5.3
**F···Am–Se**	–5.6	–5.7	–5.4	–5.5
Planar (C_{2v} / C_s)				
System	DLPNO-CCSD(T)[c]	ZORA-BLYP-D3(BJ)/TZ2P[d]	ZORA-BLYP-D3(BJ)/QZ4P	ZORA-M06-2X/TZ2P
**F···Ur–O**	–4.6	–4.9	–4.6	–5.3
**F···Ur–S**	–6.4	–6.4	–6.1	–6.9
**F···Ur–Se**	–7.1	–6.9	–6.6	–7.6
**F···Am–O**	–4.4	–4.5	–4.3	–4.3
**F···Am–S**	–5.1	–5.1	–4.9	–4.9
**F···Am–Se**	–5.3	–5.3	–5.1	–5.1

[a] Bond energies are with respect to the monomers in their equilibrium geometry
[b] Optimization with constrained C_{2v} (**F···Ur–X**) or C_s (**F···Am–X**) symmetry.
[c] Computations performed using TightPNO and a def2-QZVPP basis set.
[d] Level of theory used in this work.

Table S2. Decomposition of the bond energy ΔE_{bond} (in kcal mol^{-1}) of the hydrogen-bond interaction in the **F···Ur–X** and **F···Am–X** C_1 (non-planar) equilibrium complexes with X = O, S, and Se.[a]

All ZORA-BLYP-D3(BJ)/TZ2P (used in this work)							
System	ΔE_{bond}	ΔE_{strain}	ΔE_{int}	ΔE_{Pauli}	ΔV_{elstat}	ΔE_{oi}	ΔE_{disp}
**F···Ur–O**	–5.2	0.4	–5.7	5.7	–7.0	–2.8	–1.6
**F···Ur–S**	–6.4	0.5	–6.8	7.1	–8.5	–3.7	–1.7
**F···Ur–Se**	–6.9	0.3	–7.2	7.8	–9.1	–4.1	–1.8
**F···Am–O**	–4.9	0.1	–5.0	6.7	–6.7	–3.4	–1.7
**F···Am–S**	–5.5	0.1	–5.6	7.5	–7.4	–3.9	–1.8
**F···Am–Se**	–5.7	0.1	–5.8	7.8	–7.6	–4.0	–1.9
ZORA-BLYP-D3(BJ)/QZ4P//ZORA-BLYP-D3(BJ)/TZ2P							
System	ΔE_{bond}	ΔE_{strain}	ΔE_{int}	ΔE_{Pauli}	ΔV_{elstat}	ΔE_{oi}	ΔE_{disp}
**F···Ur–O**	–5.0	0.4	–5.4	5.7	–7.0	–2.5	–1.6
**F···Ur–S**	–6.1	0.5	–6.6	7.1	–8.5	–3.4	–1.7
**F···Ur–Se**	–6.6	0.3	–6.9	7.8	–9.1	–3.8	–1.8
**F···Am–O**	–4.7	0.1	–4.8	7.0	–6.8	–3.3	–1.7
**F···Am–S**	–5.2	0.1	–5.4	7.8	–7.6	–3.8	–1.8
**F···Am–Se**	–5.4	0.2	–5.6	8.0	–7.8	–3.9	–1.9
ZORA-M06-2X/TZ2P//ZORA-BLYP-D3(BJ)/TZ2P							
System	ΔE_{bond}	ΔE_{strain}	ΔE_{int}	ΔE_{Pauli}	ΔV_{elstat}	ΔE_{oi}	ΔE_{disp}
**F···Ur–O**	–5.5	0.5	–6.0	3.5	–7.3	–2.3	–
**F···Ur–S**	–6.9	0.2	–7.1	4.8	–8.9	–3.0	–
**F···Ur–Se**	–7.5	0.0	–7.5	5.3	–9.5	–3.3	–
**F···Am–O**	–4.7	0.3	–4.9	4.5	–6.9	–2.6	–
**F···Am–S**	–5.3	0.3	–5.6	5.1	–7.6	–3.0	–
**F···Am–Se**	–5.5	0.3	–5.8	5.2	–7.9	–3.1	–

[a] Bond energies are with respect to the monomers in their equilibrium geometry

Table S3. Decomposition of the bond energy ΔE_{bond} (in kcal mol^{-1}) of the hydrogen-bond interaction in the **F···Ur–X** and **F···Am–X** planar complexes with X = O, S, and Se.[a,b]

All ZORA-BLYP-D3(BJ)/TZ2P (used in this work)									
System	ΔE_{bond}	ΔE_{strain}	ΔE_{int}	ΔE_{Pauli}	ΔV_{elstat}	ΔE_{oi}	ΔE_{σ}	ΔE_{π}	ΔE_{disp}
**F···Ur–O**	–4.9	1.1	–6.0	6.0	–7.4	–3.0	–2.7	–0.3	–1.6
**F···Ur–S**	–6.4	0.5	–6.9	7.1	–8.6	–3.7	–3.2	–0.5	–1.7
**F···Ur–Se**	–6.9	0.3	–7.2	7.6	–9.0	–4.0	–3.5	–0.5	–1.8
**F···Am–O**	–4.5	0.1	–4.6	5.6	–6.1	–3.0	–2.8	–0.2	–1.1
**F···Am–S**	–5.1	0.1	–5.2	6.4	–6.8	–3.5	–3.2	–0.3	–1.2
**F···Am–Se**	–5.3	0.1	–5.4	6.8	–7.2	–3.8	–3.4	–0.3	–1.2
ZORA-BLYP-D3(BJ)/QZ4P//ZORA-BLYP-D3(BJ)/TZ2P									
System	ΔE_{bond}	ΔE_{strain}	ΔE_{int}	ΔE_{Pauli}	ΔV_{elstat}	ΔE_{oi}	ΔE_{σ}	ΔE_{π}	ΔE_{disp}
**F···Ur–O**	–4.6	1.2	–5.8	6.0	–7.4	–2.8	–2.4	–0.3	–1.6
**F···Ur–S**	–6.1	0.6	–6.6	7.1	–8.6	–3.4	–3.0	–0.5	–1.7
**F···Ur–Se**	–6.6	0.3	–7.0	7.6	–9.1	–3.8	–3.2	–0.6	–1.8
**F···Am–O**	–4.3	0.1	–4.4	5.7	–6.2	–2.8	–2.6	–0.2	–1.1
**F···Am–S**	–4.9	0.1	–4.9	6.5	–7.0	–3.3	–3.0	–0.3	–1.2
**F···Am–Se**	–5.1	0.1	–5.2	6.9	–7.3	–3.6	–3.2	–0.3	–1.2
ZORA-M06-2X/TZ2P//ZORA-BLYP-D3(BJ)/TZ2P									
System	ΔE_{bond}	ΔE_{strain}	ΔE_{int}	ΔE_{Pauli}	ΔV_{elstat}	ΔE_{oi}	ΔE_{σ}	ΔE_{π}	Correction[c]
**F···Ur–O**	–5.3	1.1	–6.3	3.8	–7.7	–2.4	–2.7	0.0	0.3
**F···Ur–S**	–6.9	0.3	–7.2	4.8	–8.9	–3.0	–3.3	0.0	0.3
**F···Ur–Se**	–7.6	0.0	–7.5	5.2	–9.5	–3.2	–3.4	–0.1	0.3
**F···Am–O**	–4.3	0.2	–4.5	4.1	–6.3	–2.3	–2.6	0.0	0.3
**F···Am–S**	–4.9	0.2	–5.1	4.8	–7.1	–2.8	–3.1	0.0	0.3
**F···Am–Se**	–5.1	0.2	–5.3	5.1	–7.5	–2.9	–3.2	0.0	0.3

[a] Optimized at ZORA-BLYP-D3(BJ)/TZ2P with constrained C_{2v} (**F···Ur–X**) or C_s (**F···Am–X**) symmetry.
[b] Bond energies are with respect to the monomers in their equilibrium geometry
[c] For the meta-hybrid functional M06-2X, the orbital interaction (ΔE_{oi}) is split in symmetry contributions by the so-called Transition State procedure which uses an approximate Fock operator (see for details: T. Ziegler, A. Rauk, *Theoretica Chimica Acta* **1977**, *46*, 1–10; www.scm.com). The correction term adds a correction such that the total ΔE_{oi} is the correct one.

Table S4. Gross Mulliken populations (in electrons) and orbital overlap (S) of the molecular orbitals (MOs) involved in the hydrogen-bond interaction in the **F···Ur–X** and **F···Am–X** planar complexes with X = O, S, and Se.[a,b]

	F		Ur–X		
System	MO	Gross population	MO	Gross population	Overlap (S)
**F···Ur–O**	σ_{HOMO}	1.986	σ_{LUMO+1}	0.006	0.11
	σ_{HOMO-1}	1.997	σ_{LUMO}	0.009	0.25
**F···Ur–S**	σ_{HOMO}	1.984	σ_{LUMO+1}	0.008	0.13
	σ_{HOMO-1}	1.995	σ_{LUMO}	0.011	0.26
**F···Ur–Se**	σ_{HOMO}	1.983	σ_{LUMO+1}	0.010	0.13
	σ_{HOMO-1}	1.995	σ_{LUMO}	0.012	0.27
	**F**		**Am–X**		
System	MO	Gross population	MO	Gross population	Overlap (S)
**F···Am–O**	σ_{HOMO}	1.985	σ_{LUMO}	0.016	0.08
**F···Am–S**	σ_{HOMO}	1.983	σ_{LUMO}	0.019	0.08
**F···Am–Se**	σ_{HOMO}	1.982	σ_{LUMO}	0.020	0.08

[a] Optimized at ZORA-BLYP-D3(BJ)/TZ2P with constrained C_{2v} (**F···Ur–X**) or C_s (**F···Am–X**) symmetry.
[b] See Figure 5 for the MO interaction diagrams.

Table S5. Orbital energies ε (in eV) of the molecular orbitals (MOs) resulting from the hydrogen-bond interaction in the **F···Ur–X** and **F···Am–X** planar complexes with X = O, S, and Se.[a,b]

System	MO	ε	MO	ε
**F···Ur–O**	σ_1	–11.75	σ_1*	0.09
	σ_2	–7.21	σ_2*	0.28
**F···Ur–S**	σ_1	–11.92	σ_1*	–0.07
	σ_2	–7.39	σ_2*	0.15
**F···Ur–Se**	σ_1	–11.99	σ_1*	–0.13
	σ_2	–7.42	σ_2*	0.10
**F···Am–O**	σ_3	–7.06	σ_3*	0.15
**F···Am–S**	σ_3	–7.18	σ_3*	0.04
**F···Am–Se**	σ_3	–7.22	σ_3*	0.00

[a] Optimized at ZORA-BLYP-D3(BJ)/TZ2P with constrained C_{2v} (**F···Ur–X**) or C_s (**F···Am–X**) symmetry.
[b] See Figure 5 for the MO interaction diagrams.

Table S6. Orbital energies ε (in eV) and orbital overlap of fragment molecular orbitals (FMOs), and the molecular orbitals (MOs) resulting from the π interaction upon C–N bond formation in **Am–X** (with X = O, S, and Se).[a,b]

System	FMO[c]	ε	$\langle N_{LP} \mid \ldots \rangle$	MO	ε
**Am–O**	N_{LP}	–7.32	–	π_1	–9.86
	$\pi_{C=O}$	–8.95	0.15	π_2	–6.69
	$\pi^*_{C=O}$	–2.50	0.29	π_3	–0.75
**Am–S**	N_{LP}	–7.65	–	π_1	–9.28
	$\pi_{C=S}$	–7.16	0.18	π_2	–5.80
	$\pi^*_{C=S}$	–3.56	0.26	π_3	–1.99
**Am–Se**	N_{LP}	–7.76	–	π_1	–9.24
	$\pi_{C=Se}$	–6.61	0.18	π_2	–5.44
	$\pi^*_{C=Se}$	–3.76	0.25	π_3	–2.16

[a] Computed at ZORA-BLYP-D3(BJ)/TZ2P.
[b] See Figure 7 for the MO interaction diagrams.
[c] The FMO energies are calculated within the final molecular density, see Method S4.

Table S7. Decomposition of the interaction energy ΔE_{int} (in kcal mol^{-1}) upon C–N bond formation in **Am–X** (with X = O, S, and Se).[a]

System	ΔE_{int}	ΔE_{Pauli}	ΔV_{elstat}	ΔE_{oi}	ΔE_{σ}	ΔE_{π}	ΔE_{disp}
**Am–O**	–138.3	344.9	–184.5	–296.8	–258.6	–38.3	–1.8
**Am–S**	–141.3	374.6	–196.8	–316.7	–271.8	–44.9	–2.6
**Am–Se**	–144.1	382.8	–198.1	–326.1	–278.6	–47.5	–2.7

[a] Computed using an unrestricted formalism at ZORA-BLYP-D3(BJ)/TZ2P.

Table S8. Gross Mulliken populations (in electrons), contributions (in %) to the $\pi_{C=X}$ and $\pi^*_{C=X}$ MOs, and orbital overlap of the p atomic orbitals involved in the π C=X bond formation in **Al–X** (with X = O, S, and Se).[a,b]

	••CH_2				••X				
System	FMO	Gross population	%$\pi_{C=X}$	%$\pi^*_{C=X}$	FMO	Gross population	%$\pi_{C=X}$	%$\pi^*_{C=X}$	$\langle$C-2p | X-np$\rangle$
**Al–O**	C-2p	0.67	33	69	O-2p	1.29	65	35	0.31
**Al–S**	C-2p	0.85	43	56	S-3p	1.13	56	46	0.30
**Al–Se**	C-2p	0.85	43	54	Se-4p	1.13	56	46	0.28

[a] Computed at ZORA-BLYP-D3(BJ)/TZ2P.
[b] See Figure 8 for the orbital interaction diagram.

Table S9. Decomposition of the interaction energy ΔE_{int} (in kcal mol^{-1}), p orbital overlap, and the energy of the C=X π* level (in eV), of the ··CH_2 fragment interacting with the chalcogen atoms (··X) in **Al–X** at the equilibrium *d*(C=X) of **Al–O**, **Al–S**, and **Al–Se**.[a,b]

d(C=X)	X	ΔE_{int}	ΔE_{Pauli}	ΔV_{elstat}	ΔE_{oi}	ΔE_{disp}	⟨C-2p| X-np⟩	$\varepsilon(\pi^*_{C=X})$
1.211	O	–191.5	465.3	–192.8	–463.3	–0.8	0.31	–2.6
	S	31.0	876.0	–368.1	–474.9	–2.0	0.47	–2.0
	Se	201.6	1181.6	–526.8	–451.0	–2.2	0.49	–1.8
1.621	O	–124.8	117.7	–60.9	–180.9	–0.8	0.18	–4.7
	S	–138.3	270.6	–155.5	–251.5	–2.0	0.30	–3.5
	Se	–110.0	358.9	–210.5	–256.2	–2.2	0.33	–3.2
1.771	O	–95.1	69.7	–38.4	–125.6	–0.8	0.15	–5.1
	S	–130.6	171.4	–105.6	–194.4	–1.9	0.25	–4.0
	Se	–120.3	228.2	–143.2	–203.1	–2.2	0.28	–3.6

[a] The equilibrium *d*(C=X) are 1.211 (X = O), 1.621 (X = S), and 1.771 (X = Se) Å. All other bond distances and angles were frozen in the equilibrium geometry of the respecting **Al–X**.
[b] Computed using an unrestricted formalism at ZORA-BLYP-D3(BJ)/TZ2P.

Table S10. Decomposition of the interaction energy ΔE_{int} (in kcal mol^{-1}) of the hydrogen-bond interaction in **F···Ur–X** (with X = O, S, and Se) as function of the hydrogen-bond distance O···(H)N (in Å).[a]

System	O···(H)N	ΔE_{int}	ΔE_{Pauli}	ΔV_{elstat}	ΔE_{oi}	ΔE_{disp}
			Constant method[b]			
**F···Ur–O**	2.95	–5.53	11.02	–9.97	–4.66	–1.93
	3.00	–5.79	9.29	–9.13	–4.12	–1.84
	3.05	–5.94	7.83	–8.38	–3.64	–1.75
	3.10	–6.01	6.60	–7.71	–3.22	–1.67
	3.15	–6.01	5.56	–7.13	–2.86	–1.59
	3.20	–5.97	4.69	–6.60	–2.54	–1.51
	3.25	–5.88	3.97	–6.15	–2.26	–1.44
**F···Ur–S**	2.95	–6.59	11.48	–10.84	–5.25	–1.97
	3.00	–6.78	9.69	–9.95	–4.64	–1.88
	3.05	–6.88	8.18	–9.16	–4.11	–1.80
	3.10	–6.91	6.90	–8.46	–3.64	–1.71
	3.15	–6.87	5.82	–7.83	–3.23	–1.63
	3.20	–6.79	4.91	–7.28	–2.87	–1.55
	3.25	–6.67	4.16	–6.79	–2.56	–1.47
**F···Ur–Se**	2.95	–6.95	11.56	–11.08	–5.44	–1.99
	3.00	–7.11	9.77	–10.18	–4.80	–1.90
	3.05	–7.18	8.25	–9.38	–4.25	–1.81
	3.10	–7.18	6.97	–8.67	–3.76	–1.72

System	O···(H)N	ΔE_{int}	ΔE_{Pauli}	ΔV_{elstat}	ΔE_{oi}	ΔE_{disp}
	3.15	−7.12	5.89	−8.04	−3.34	−1.64
	3.20	−7.02	4.99	−7.49	−2.97	−1.56
	3.25	−6.89	4.22	−6.98	−2.64	−1.48
			Variational method[c]			
**F···Ur–O**	2.95	−5.17	10.37	−9.34	−4.23	−1.96
	3.00	−5.43	8.85	−8.64	−3.80	−1.84
	3.05	−5.58	7.47	−7.94	−3.37	−1.75
	3.10	−5.65	6.31	−7.31	−2.99	−1.67
	3.15	−5.65	5.33	−6.75	−2.65	−1.58
	3.20	−5.61	4.50	−6.25	−2.35	−1.50
	3.25	−5.52	3.80	−5.80	−2.09	−1.43
**F···Ur–S**	2.95	−6.55	11.35	−10.75	−5.18	−1.98
	3.00	−6.74	9.63	−9.88	−4.60	−1.89
	3.05	−6.84	8.13	−9.10	−4.07	−1.80
	3.10	−6.84	6.87	−8.40	−3.60	−1.71
	3.15	−6.80	5.79	−7.77	−3.20	−1.63
	3.20	−6.70	4.90	−7.21	−2.84	−1.55
	3.25	−6.58	4.14	−6.72	−2.52	−1.47
**F···Ur–Se**	2.95	−6.96	11.50	−11.05	−5.41	−2.00
	3.00	−7.09	9.73	−10.13	−4.78	−1.91
	3.05	−7.16	8.25	−9.35	−4.24	−1.82
	3.10	−7.15	6.97	−8.64	−3.75	−1.73
	3.15	−7.10	5.89	−8.02	−3.33	−1.64
	3.20	−6.97	4.99	−7.44	−2.96	−1.57
	3.25	−6.85	4.23	−6.96	−2.64	−1.49

[a] All energies are computed at ZORA-BLYP-D3(BJ)/TZ2P.
[b] The O···(H)N distance was varied in a stepwise manner (step size of 0.05 Å) while the geometries of the interacting molecules were frozen in the planar C_{2v} equilibrium geometry.
[c] The O···(H)N distance was varied in a stepwise manner (step size of 0.05 Å) while the geometries of the two interacting molecules were allowed to relax without constraints at each step.

Table S11. List with Cartesian coordinates (in Å), total bond energies E (in kcal mol^{-1}), and number of imaginary frequencies (N_{imag}) of all geometries optimized at ZORA-BLYP-D3(BJ)/TZ2P. It is specified if no symmetry (C_1) or planar symmetry (C_s or C_{2v}) was enforced during the optimization process.

Formaldehyde (Form or Al–O) C_1/C_{2v}

E (ZORA-BLYP-D3(BJ)/TZ2P): −494.4
E (ZORA-BLYP-D3(BJ)/QZ4P//ZORA-BLYP-D3(BJ)/TZ2P): −495.6
E (ZORA-M06-2X/TZ2P//ZORA-BLYP-D3(BJ)/TZ2P): −750.7
E (DLPNO-CCSD(T)/def2-QZVPP//ZORA-BLYP-D3(BJ)/TZ2P): −71766.7
N_{imag} = 0

1.O	0.000000	0.000000	4.717179
2.C	0.000000	0.000000	5.928622
3.H	0.000000	0.945278	6.519261
4.H	0.000000	−0.945278	6.519261

Thialdehyde (Al–S) C_1/C_{2v}

E (ZORA-BLYP-D3(BJ)/TZ2P): −420.1
N_{imag} = 0

1.S	0.000000	0.000000	0.145806
2.C	0.000000	0.000000	1.766846
3.H	0.926805	0.000000	2.349313
4.H	−0.926805	0.000000	2.349313

Selenoaldehyde (Al–Se) C_1/C_{2v}

E (ZORA-BLYP-D3(BJ)/TZ2P): −397.2
N_{imag} = 0

1.Se	0.000000	0.000000	0.136344
2.C	0.000000	0.000000	1.906872
3.H	0.928684	0.000000	2.479031
4.H	−0.928684	0.000000	2.479031

Carboxamide (Am–O) C_1/C_s

E (ZORA-BLYP-D3(BJ)/TZ2P): −783.0
E (ZORA-BLYP-D3(BJ)/QZ4P//ZORA-BLYP-D3(BJ)/TZ2P): −785.3
E (ZORA-M06-2X/TZ2P//ZORA-BLYP-D3(BJ)/TZ2P): −1182.2
E (DLPNO-CCSD(T)/def2-QZVPP//ZORA-BLYP-D3(BJ)/TZ2P): −106484.2
N_{imag} = 0

1.H	1.363758	−0.619714	0.000000
2.C	−0.339198	0.575623	0.000000
3.N	0.351717	−0.604551	0.000000
4.H	−0.156944	−1.482326	0.000000
5.O	−1.557565	0.673168	0.000000
6.H	0.338232	1.457800	0.000000

Thioamide (Am–S) C_1/C_s

E (ZORA-BLYP-D3(BJ)/TZ2P): −706.1
E (ZORA-BLYP-D3(BJ)/QZ4P//ZORA-BLYP-D3(BJ)/TZ2P): −708.8
E (ZORA-M06-2X/TZ2P//ZORA-BLYP-D3(BJ)/TZ2P): −1061.2
E (DLPNO-CCSD(T)/def2-QZVPP//ZORA-BLYP-D3(BJ)/TZ2P): −308911.2
N_{imag} = 0

1.H	1.302717	−0.867521	0.000000
2.C	−0.160730	0.597172	0.000000
3.N	0.308525	−0.670222	0.000000
4.H	−0.345934	−1.446738	0.000000
5.S	−1.748529	1.045639	0.000000
6.H	0.643951	1.341669	0.000000

Selenoamide (Am–Se) C_1/C_s

E (ZORA-BLYP-D3(BJ)/TZ2P): −683.4
E (ZORA-BLYP-D3(BJ)/QZ4P//ZORA-BLYP-D3(BJ)/TZ2P): −685.5
E (ZORA-M06-2X/TZ2P//ZORA-BLYP-D3(BJ)/TZ2P): −1033.4

E (DLPNO-CCSD(T)/def2-QZVPP//ZORA-BLYP-D3(BJ)/TZ2P): −1565604.6
N_{imag} = 0

1.N	0.651514	0.377919	0.000000
2.H	0.534189	1.387177	0.000000
3.Se	−2.153419	0.137403	0.000000
4.C	−0.433722	−0.417069	0.000000
5.H	−0.190367	−1.482577	0.000000
6.H	1.591805	−0.002853	0.000000

Urea (Ur–O) C_1

E (ZORA-BLYP-D3(BJ)/TZ2P): −1059.6
E (ZORA-BLYP-D3(BJ)/QZ4P//ZORA-BLYP-D3(BJ)/TZ2P): −1062.9
E (ZORA-M06-2X/TZ2P//ZORA-BLYP-D3(BJ)/TZ2P): −1604.7
E (DLPNO-CCSD(T)/def2-QZVPP//ZORA-BLYP-D3(BJ)/TZ2P): −141193.6
N_{imag} = 0

1.H	1.120695	1.327750	−0.247362
2.H	0.780695	−1.045059	0.221038
3.N	1.705547	−1.132912	−0.187329
4.C	2.617156	−0.093691	0.005875
5.H	2.122237	−2.053368	−0.099821
6.N	2.024200	1.157545	0.182326
7.H	2.681221	1.926445	0.106617
8.O	3.831960	−0.264860	0.019585

Thiourea (Ur–S) C_1

E (ZORA-BLYP-D3(BJ)/TZ2P): −981.0
E (ZORA-BLYP-D3(BJ)/QZ4P//ZORA-BLYP-D3(BJ)/TZ2P): −985.0
E (ZORA-M06-2X/TZ2P//ZORA-BLYP-D3(BJ)/TZ2P): −1481.5
E (DLPNO-CCSD(T)/def2-QZVPP//ZORA-BLYP-D3(BJ)/TZ2P): −343618.4
N_{imag} = 0

1.H	1.122698	1.301367	−0.243081
2.H	0.793539	−1.012648	0.268495
3.N	1.733746	−1.131178	−0.097238
4.C	2.635692	−0.097777	−0.002551
5.H	2.128280	−2.060794	−0.022901
6.N	2.063567	1.147898	0.107700
7.H	2.704260	1.927473	0.022803
8.S	4.292827	−0.334804	−0.019977

Selenourea (Ur–Se) C_1

E (ZORA-BLYP-D3(BJ)/TZ2P): −959.1
E (ZORA-BLYP-D3(BJ)/QZ4P//ZORA-BLYP-D3(BJ)/TZ2P): −962.3
E (ZORA-M06-2X/TZ2P//ZORA-BLYP-D3(BJ)/TZ2P): −1455.0
E (DLPNO-CCSD(T)/def2-QZVPP//ZORA-BLYP-D3(BJ)/TZ2P): −1600312.0
N_{imag} = 0

1.H	1.142110	1.340893	−0.177710
2.H	0.750602	−0.988126	0.186585
3.N	1.714359	−1.119219	−0.107066
4.C	2.622014	−0.106030	0.001321
5.H	2.084298	−2.060387	−0.064201
6.N	2.096147	1.148412	0.113685
7.H	2.754933	1.915440	0.067041
8.Se	4.438874	−0.409378	−0.003719

Formaldehyde–Carboxamide (F···Am–O) C_s

E (ZORA-BLYP-D3(BJ)/TZ2P): −1281.9
E (ZORA-BLYP-D3(BJ)/QZ4P//ZORA-BLYP-D3(BJ)/TZ2P): −1285.1
E (ZORA-M06-2X/TZ2P//ZORA-BLYP-D3(BJ)/TZ2P): −1937.2
E (DLPNO-CCSD(T)/def2-QZVPP//ZORA-BLYP-D3(BJ)/TZ2P): −178255.4
N_{imag} = 0

1.H	1.545418	−0.085028	0.000000
2.C	−0.501912	0.297734	0.000000
3.N	0.611695	−0.489216	0.000000
4.H	0.496985	−1.497059	0.000000

5.O	−1.658118	−0.109266	0.000000
6.H	−0.246845	1.380319	0.000000
7.C	4.498583	0.519795	0.000000
8.H	5.344433	1.240184	0.000000
9.O	3.344063	0.896136	0.000000
10.H	4.765765	−0.559819	0.000000

Formaldehyde–Thioamide (F···Am–S) C_s

E (ZORA-BLYP-D3(BJ)/TZ2P): −1205.6
E (ZORA-BLYP-D3(BJ)/QZ4P//ZORA-BLYP-D3(BJ)/TZ2P): −1209.2
E (ZORA-M06-2X/TZ2P//ZORA-BLYP-D3(BJ)/TZ2P): −1816.8
E (DLPNO-CCSD(T)/def2-QZVPP//ZORA-BLYP-D3(BJ)/TZ2P): −380683.0
N_{imag} = 0

1.H	1.595582	−0.074504	0.000000
2.C	−0.429432	0.363081	0.000000
3.N	0.646469	−0.446992	0.000000
4.H	0.500974	−1.451971	0.000000
5.S	−2.016552	−0.108745	0.000000
6.H	−0.153583	1.423711	0.000000
7.C	4.524942	0.467622	0.000000
8.H	5.378491	1.178067	0.000000
9.O	3.374768	0.858499	0.000000
10.H	4.778410	−0.614989	0.000000

Formaldehyde–Selenoamide (F···Am–Se) C_s

E (ZORA-BLYP-D3(BJ)/TZ2P): −1183.0
E (ZORA-BLYP-D3(BJ)/QZ4P//ZORA-BLYP-D3(BJ)/TZ2P): −1186.1
E (ZORA-M06-2X/TZ2P//ZORA-BLYP-D3(BJ)/TZ2P): −1789.2
E (DLPNO-CCSD(T)/def2-QZVPP//ZORA-BLYP-D3(BJ)/TZ2P): −1637376.6
N_{imag} = 0

1.H	1.612667	−0.036201	0.000000
2.C	−0.403985	0.427743	0.000000
3.N	0.656044	−0.392538	0.000000
4.H	0.497122	−1.396070	0.000000
5.Se	−2.146842	−0.072955	0.000000
6.H	−0.132106	1.486132	0.000000
7.C	4.540022	0.389169	0.000000
8.H	5.423924	1.061171	0.000000
9.O	3.408153	0.830850	0.000000
10.H	4.745071	−0.703521	0.000000

Formaldehyde–Urea (F···Ur–O) C_{2v}

E (ZORA-BLYP-D3(BJ)/TZ2P): −1558.8
E (ZORA-BLYP-D3(BJ)/QZ4P//ZORA-BLYP-D3(BJ)/TZ2P): −1563.1
E (ZORA-M06-2X/TZ2P//ZORA-BLYP-D3(BJ)/TZ2P): -2360.6
E (DLPNO-CCSD(T)/def2-QZVPP//ZORA-BLYP-D3(BJ)/TZ2P): −212964.9
N_{imag} = 2 (−183; −264 cm^{-1})

1.H	0.000000	1.164850	2.901331
2.H	0.000000	−1.164850	2.901331
3.N	0.000000	−1.163020	1.889625
4.C	0.000000	0.000000	1.142011
5.H	0.000000	−2.033836	1.380122
6.N	0.000000	1.163020	1.889625
7.H	0.000000	2.033836	1.380122
8.O	0.000000	0.000000	−0.091113
9.O	0.000000	0.000000	4.793044
10.C	0.000000	0.000000	6.007714
11.H	0.000000	0.945327	6.590889
12.H	0.000000	−0.945327	6.590889

Formaldehyde–Thiourea (F···Ur–S) C_{2v}

E (ZORA-BLYP-D3(BJ)/TZ2P): −1481.8
E (ZORA-BLYP-D3(BJ)/QZ4P//ZORA-BLYP-D3(BJ)/TZ2P): −1486.6

E (ZORA-M06-2X/TZ2P//ZORA-BLYP-D3(BJ)/TZ2P): −2239.1
E (DLPNO-CCSD(T)/def2-QZVPP//ZORA-BLYP-D3(BJ)/TZ2P): −415391.5
N_{imag} = 1 (−73 cm^{-1})

1.H	0.000000	1.149063	2.900154
2.H	0.000000	−1.149063	2.900154
3.N	0.000000	−1.151722	1.885810
4.C	0.000000	0.000000	1.154619
5.H	0.000000	−2.024798	1.379629
6.N	0.000000	1.151722	1.885810
7.H	0.000000	2.024798	1.379629
8.S	0.000000	0.000000	−0.531409
9.O	0.000000	0.000000	4.755014
10.C	0.000000	0.000000	5.970826
11.H	0.000000	0.945354	6.552677
12.H	0.000000	−0.945354	6.552677

Formaldehyde–Selenourea (F···Ur–Se) C_{2v}

E (ZORA-BLYP-D3(BJ)/TZ2P): −1460.3
E (ZORA-BLYP-D3(BJ)/QZ4P//ZORA-BLYP-D3(BJ)/TZ2P): −1464.5
E (ZORA-M06-2X/TZ2P//ZORA-BLYP-D3(BJ)/TZ2P): −2213.2
E (DLPNO-CCSD(T)/def2-QZVPP//ZORA-BLYP-D3(BJ)/TZ2P): −1672085.9
N_{imag} = 0

1.H	0.000000	1.156092	2.920045
2.H	0.000000	−1.156092	2.920045
3.N	0.000000	−1.151519	1.904426
4.C	0.000000	0.000000	1.187633
5.H	0.000000	−2.021749	1.392413
6.N	0.000000	1.151519	1.904426
7.H	0.000000	2.021749	1.392413
8.Se	0.000000	0.000000	−0.666516
9.O	0.000000	0.000000	4.754431
10.C	0.000000	0.000000	5.971201
11.H	0.000000	0.945281	6.552537
12.H	0.000000	−0.945281	6.552537

Formaldehyde–Carboxamide (F···Am–O) C_1

E (ZORA-BLYP-D3(BJ)/TZ2P): −1282.3
E (ZORA-BLYP-D3(BJ)/QZ4P//ZORA-BLYP-D3(BJ)/TZ2P): −1285.5
E (ZORA-M06-2X/TZ2P//ZORA-BLYP-D3(BJ)/TZ2P): −1937.6
E (DLPNO-CCSD(T)/def2-QZVPP//ZORA-BLYP-D3(BJ)/TZ2P): −178255.7
N_{imag} = 0

1.H	1.748838	−0.384188	0.534904
2.C	−0.006890	0.328262	−0.324357
3.N	0.814155	−0.621150	0.205965
4.H	0.467347	−1.568986	0.307742
5.O	−1.147233	0.140284	−0.731389
6.H	0.481306	1.328563	−0.356186
7.C	4.090439	0.685224	−0.414168
8.H	5.117873	1.093444	−0.518636
9.O	3.659557	0.316925	0.661004
10.H	3.478006	0.630129	−1.340238

Formaldehyde–Thioamide (F···Am–S) C_1

E (ZORA-BLYP-D3(BJ)/TZ2P): −1206.0
E (ZORA-BLYP-D3(BJ)/QZ4P//ZORA-BLYP-D3(BJ)/TZ2P): −1209.6
E (ZORA-M06-2X/TZ2P//ZORA-BLYP-D3(BJ)/TZ2P): −1817.2
E (DLPNO-CCSD(T)/def2-QZVPP//ZORA-BLYP-D3(BJ)/TZ2P): −380683.3
N_{imag} = 0

1.H	1.778138	−0.439270	0.536396
2.C	0.072126	0.362880	−0.315205
3.N	0.849048	−0.629754	0.158381
4.H	0.504569	−1.584283	0.122721
5.S	−1.433297	0.209397	−0.985701
6.H	0.546517	1.346432	−0.213678

7.C	4.100843	0.675109	−0.331032
8.H	5.125188	1.097887	−0.395635
9.O	3.649607	0.263880	0.720628
10.H	3.510660	0.646230	−1.272235

Formaldehyde–Selenoamide (F···Am–Se) ***C***

Formaldehyde–Selenoamide (F···Am–Se) C_1

E (ZORA-BLYP-D3(BJ)/TZ2P): −1183.4
E (ZORA-BLYP-D3(BJ)/QZ4P//ZORA-BLYP-D3(BJ)/TZ2P): −1186.5
E (ZORA-M06-2X/TZ2P//ZORA-BLYP-D3(BJ)/TZ2P): −1789.6
E (DLPNO-CCSD(T)/def2-QZVPP//ZORA-BLYP-D3(BJ)/TZ2P): −1637376.9
N_{imag} = 0

1.H	1.810844	−0.485657	0.556593
2.C	0.125763	0.392243	−0.247062
3.N	0.865111	−0.633222	0.198472
4.H	0.484551	−1.574062	0.153117
5.Se	−1.538517	0.300192	−0.958644
6.H	0.626848	1.358596	−0.145351
7.C	4.086009	0.652065	−0.394809
8.H	5.101493	1.088805	−0.494466
9.O	3.681749	0.223277	0.669070
10.H	3.459547	0.626270	−1.312279

Formaldehyde–Urea (F···Ur–O) C_1

E (ZORA-BLYP-D3(BJ)/TZ2P): −1559.2
E (ZORA-BLYP-D3(BJ)/QZ4P//ZORA-BLYP-D3(BJ)/TZ2P): −1563.5
E (ZORA-M06-2X/TZ2P//ZORA-BLYP-D3(BJ)/TZ2P): -2360.9
E (DLPNO-CCSD(T)/def2-QZVPP//ZORA-BLYP-D3(BJ)/TZ2P): −212965.6
N_{imag} = 0

1.C	2.668797	−0.097690	0.047272
2.H	2.158515	−2.060463	−0.070054
3.N	2.071702	1.150472	0.184226
4.H	2.712042	1.931525	0.119242
5.O	3.883054	−0.270202	0.152245
6.H	1.125947	1.296161	−0.152639
7.H	0.788567	−1.001855	−0.050819
8.N	1.780745	−1.133249	−0.217136
9.H	−2.607569	1.575983	−0.259647
10.O	−0.946297	0.409966	−0.173954
11.C	−2.146299	0.578695	−0.094194
12.H	−2.838542	−0.256094	0.147578

Formaldehyde–Thiourea (F···Ur–S) C_1

E (ZORA-BLYP-D3(BJ)/TZ2P): −1481.8
E (ZORA-BLYP-D3(BJ)/QZ4P//ZORA-BLYP-D3(BJ)/TZ2P): −1486.6
E (ZORA-M06-2X/TZ2P//ZORA-BLYP-D3(BJ)/TZ2P): -2239.1
E (DLPNO-CCSD(T)/def2-QZVPP//ZORA-BLYP-D3(BJ)/TZ2P): −415391.6
N_{imag} = 0

1.H	0.493525	1.331477	−0.088354
2.H	0.249924	−0.951947	−0.142831
3.N	1.249773	−1.061242	−0.011096
4.C	2.101311	0.004850	−0.000026
5.H	1.658551	−1.983754	0.018245
6.N	1.502575	1.226340	−0.120648
7.H	2.094539	2.038997	−0.026634
8.S	3.771005	−0.173504	0.141948
9.O	−1.471890	0.414448	−0.089183
10.C	−2.666870	0.582154	0.058998
11.H	−3.098517	1.594352	0.207622
12.H	−3.379219	−0.269370	0.052421

Formaldehyde–Selenourea (F···Ur–Se) C_1

E (ZORA-BLYP-D3(BJ)/TZ2P): −1460.3
E (ZORA-BLYP-D3(BJ)/QZ4P//ZORA-BLYP-D3(BJ)/TZ2P): −1464.5
E (ZORA-M06-2X/TZ2P//ZORA-BLYP-D3(BJ)/TZ2P): -2213.2
E (DLPNO-CCSD(T)/def2-QZVPP//ZORA-BLYP-D3(BJ)/TZ2P): −1672085.9

N_{imag} = 0

1.H	0.492583	1.343511	−0.125875
2.H	0.249860	−0.953734	−0.117029
3.N	1.258251	−1.056016	−0.051892
4.C	2.090162	0.014167	−0.000301
5.H	1.672014	−1.974930	0.011903
6.N	1.501446	1.233557	−0.080556
7.H	2.098841	2.045693	−0.023241
8.Se	3.926762	−0.178159	0.162475
9.O	−1.446452	0.404895	−0.094541
10.C	−2.643265	0.562846	0.057822
11.H	−3.089065	1.575316	0.148939
12.H	−3.340976	−0.298529	0.112702
